# Estimated clinical impact of the Xpert MTB/RIF Ultra cartridge for diagnosis of pulmonary tuberculosis: A modeling study

**DOI:** 10.1371/journal.pmed.1002472

**Published:** 2017-12-14

**Authors:** Emily A. Kendall, Samuel G. Schumacher, Claudia M. Denkinger, David W. Dowdy

**Affiliations:** 1 Division of Infectious Diseases, Johns Hopkins University School of Medicine, Baltimore, Maryland, United States of America; 2 Tuberculosis Program, FIND, Geneva, Switzerland; 3 Department of Epidemiology, Johns Hopkins Bloomberg School of Public Health, Baltimore, Maryland, United States of America; Centers for Disease Control and Prevention, UNITED STATES

## Abstract

**Background:**

The Xpert MTB/RIF (Xpert) assay offers rapid and accurate diagnosis of tuberculosis (TB) but still suffers from imperfect sensitivity. The newer Xpert MTB/RIF Ultra cartridge has shown improved sensitivity in recent field trials, but at the expense of reduced specificity. The clinical implications of switching from the existing Xpert cartridge to the Xpert Ultra cartridge in different populations remain uncertain.

**Methods and findings:**

We developed a Markov microsimulation model of hypothetical cohorts of 100,000 individuals undergoing diagnostic sputum evaluation with Xpert for suspected pulmonary TB, in each of 3 emblematic settings: an HIV clinic in South Africa, a public TB center in India, and an adult primary care setting in China. In each setting, we used existing data to project likely diagnostic results, treatment decisions, and ultimate clinical outcomes, assuming use of the standard Xpert versus Xpert Ultra cartridge. Our primary outcomes were the projected number of additional unnecessary treatments generated, the projected number of TB deaths averted, and the projected number of unnecessary treatments generated per TB death averted, if standard Xpert were switched to Xpert Ultra. We also simulated alternative approaches to interpreting positive results of the Ultra cartridge’s semi-quantitative trace call. Extensive sensitivity and uncertainty analyses were performed to evaluate the drivers and generalizability of projected results. In the Indian TB center setting, replacing the standard Xpert cartridge with the Xpert Ultra cartridge was projected to avert 0.5 TB deaths (95% uncertainty range [UR]: 0, 1.3) and generate 18 unnecessary treatments (95% UR: 10, 29) per 1,000 individuals evaluated—resulting in a median ratio of 38 incremental unnecessary treatments added by Ultra per incremental death averted by Ultra compared to outcomes using standard Xpert (95% UR: 12, indefinite upper bound). In the South African HIV care setting—where TB mortality rates are higher and Ultra’s improved sensitivity has greater absolute benefit—this ratio improved to 7 unnecessary treatments per TB death averted (95% UR: 2, 43). By contrast, in the Chinese primary care setting, this ratio was much less favorable, at 372 unnecessary treatments per TB death averted (95% UR: 75, indefinite upper bound), although the projected number of unnecessary treatments using Xpert Ultra was lower (with a possibility of no increased overtreatment) when using specificity data only from lower-burden settings. Alternative interpretations of the trace call had little effect on these ratios. Limitations include uncertainty in key parameters (including the clinical implications of false-negative results), the exclusion of transmission effects, and restriction of this analysis to adult pulmonary TB.

**Conclusions:**

Switching from the standard Xpert cartridge to the Xpert Ultra cartridge for diagnosis of adult pulmonary TB may have different consequences in different clinical settings. In settings with high TB and HIV prevalence, Xpert Ultra is likely to offer considerable mortality benefit, whereas in lower-prevalence settings, Xpert Ultra will likely result in considerable overtreatment unless the possibility of higher specificity of Ultra in lower-prevalence settings in confirmed. The ideal use of the Ultra cartridge may therefore involve a more nuanced, setting-specific approach to implementation, with priority given to populations in which the anticipated prevalence of TB (and HIV) is the highest.

## Abstract 2

In this modelling study, Emily Kendall and colleagues estimate the potential for increased tuberculosis case-finding as well as unnecessary treatments when adopting the more sensitive Xpert Ultra cartridges for detecting TB in settings of high and low prevalence.

## Author summary

**Why was this study done?:**

- Xpert Ultra is a new version of a widely used molecular test for tuberculosis (TB) that has a better ability to detect TB (higher sensitivity) but also more frequently gives false-positive results (lower specificity).
- These differences in sensitivity and specificity will have different clinical implications in settings with different characteristics such as higher or lower TB and HIV prevalence among the people being tested.
- The relative advantages and disadvantages of adopting Xpert Ultra are therefore likely to differ across different clinical contexts.

**What did the researchers do and find?:**

- We modeled and compared the likely clinical outcomes, including number of TB deaths and number of unnecessary treatments, when using Xpert Ultra versus the standard Xpert assay.
- We performed this comparison for 3 different hypothetical patient populations in different medium- to high-TB-burden settings (a South African HIV clinic, an Indian TB center, and a Chinese primary care clinic).
- We found that the estimated clinical impact of switching from standard Xpert to Xpert Ultra differed dramatically between settings: Ultra yielded fewer than 10 additional unnecessary TB treatments per TB death prevented in the HIV clinic setting in South Africa, in contrast to more than 300 additional unnecessary TB treatments per TB death prevented in the general primary care setting in China.

**What do these findings mean?:**

- Xpert Ultra is likely to provide a large clinical benefit over standard Xpert in patient populations with high TB prevalence, high HIV prevalence, and high case fatality ratios for untreated TB.
- In populations with low TB prevalence or small proportions of HIV-associated or smear-negative TB, Xpert Ultra will require more cautious implementation and interpretation to avoid costly and harmful overdiagnosis of TB.

## Introduction

Introduced in 2010, Xpert MTB/RIF (Xpert)—a molecular assay for the detection of tuberculosis (TB) and resistance to rifampin—provides substantial improvements in sensitivity over sputum smear microscopy, previously the cornerstone of TB diagnosis [1]. The sensitivity of Xpert remains imperfect, however, particularly in patients with paucibacillary TB disease (often seen in the context of HIV) [2,3]. More recently, a novel cartridge, the Xpert MTB/RIF Ultra cartridge (“Xpert Ultra”), was developed for TB diagnosis using the same GeneXpert platform, but with technical enhancements (including larger specimen volume, probes for repeated elements in the mycobacterial genome, and optimized fluidics and polymerase chain reaction cycling) designed to further increase the sensitivity of Xpert for detection of TB [4]. The performance of the Xpert Ultra cartridge was subsequently evaluated in a large 10-site, 8-country study [5], which confirmed its increased sensitivity for diagnosis of active pulmonary TB relative to the existing Xpert (G4) cartridge (“standard Xpert”), using sputum culture as a reference standard. In particular, Ultra was estimated to add 5% to the sensitivity of standard Xpert among all culture-positive study participants and 13% (increasing the sensitivity for TB detection from 77% to 90%) among those infected with HIV. However, these study data also suggested a loss of specificity with Ultra, particularly among individuals with a history of previous TB treatment; false-positives increased more than 2-fold with Ultra compared to standard Xpert in those with no prior TB and more than 3-fold in those with a history of TB.

Based on its improved sensitivity, the World Health Organization has endorsed the new Ultra cartridge [6], and it has been made available to eligible countries at the same concessional pricing as the standard Xpert cartridge [7]. In deciding how best to implement use of the Ultra cartridge for diagnosis of adult pulmonary TB, it is important to consider how this trade-off between sensitivity and specificity would translate into clinical and/or public health outcomes. We therefore constructed a simulation model to explore the downstream clinical consequences of replacing the standard Xpert cartridge with the Ultra cartridge as the initial diagnostic test for presumptive pulmonary TB in 3 emblematic settings.

## Methods

### Model description and simulated settings

We developed a Markov microsimulation model of TB, using cohorts of adults (≥15 years old) undergoing diagnostic sputum evaluation for suspected pulmonary TB in a setting with Xpert capacity. Our primary comparison was of expected diagnostic and clinical outcomes using standard Xpert versus Xpert Ultra. We selected 3 emblematic settings to illustrate a range of different patient populations in which Xpert might be used: a TB diagnosis and treatment center in India’s public health sector, an ambulatory HIV care setting in South Africa, and a primary care setting in China. As detailed in Table 1, these settings differ in demographic makeup, underlying TB prevalence, HIV prevalence, prevalence of rifampin resistance, empiric treatment practices, and TB treatment outcomes. The breakdown of the resulting cohorts according to TB, HIV, and rifampin-resistance status is shown in S2 Table.

**Table 1:** Setting-specific parameters.

Parameter	Indian TB center setting	South African HIV care setting	Chinese primary care setting
***Parameters defining setting-specific cohort***^***a***^			
**TB prevalence among those tested with Xpert**	12% [1]	12% [8]	6% (assumed)
**History of previous TB**			
True TB cases	22% [9]	8% [9]	3.7% [9]
No underlying TB	14%^b^	7%^b^	3.1%^b^
**Rifampin resistance among TB cases**			
New cases	2.5% [10]	3.5% [10]	6.6% [10]
Previously treated cases	16% [10]	7.1% [10]	30% [10]
**HIV prevalence among those tested with Xpert**	5% [10]	100% (assumed)	3% [10]
**Age in years, mean (SD)**	39 (18) [9]	37 (14) [9]	46 (19) [9]
**Female (%)**	33% [9]	42% [9]	31% [9]
***Setting-specific treatment practices and outcomes***^***a***^			
Probability of empiric TB treatment if negative Xpert result	4% (2%–8%) [11,12]	40% (20%–60%) [13,14]	0% (assumed)
Case fatality ratio for drug-susceptible TB^c^	18% (10%–40%) [10]	21% (10%–50%) [10]	5% (4%–7%) [10]
Non-TB mortality rate	Dependent on setting, age, sex, and HIV status (detailed in S1 Supplemental Methods)		

^a^Cohort-defining parameters were kept fixed in the primary analysis (with ranges, when shown, used only in sensitivity analyses), whereas parameters for treatment practices and outcomes were sampled for each simulation from the triangular distribution defined by the mode and range shown.

^b^Mean of the prevalence of previous TB among notified cases and an estimated prevalence of previous TB in the overall national adult population; estimation is described in more detail in S1 Supplemental Methods.

^c^Calculated as reported mortality/incidence for 2015. Because of limited setting-specific estimates for rifampin-resistant TB case fatality ratios, a single global estimate was used (shown in Table 2).

TB, tuberculosis; Xpert, Xpert MTB/RIF.

### Model analyses

**Fig 1:**
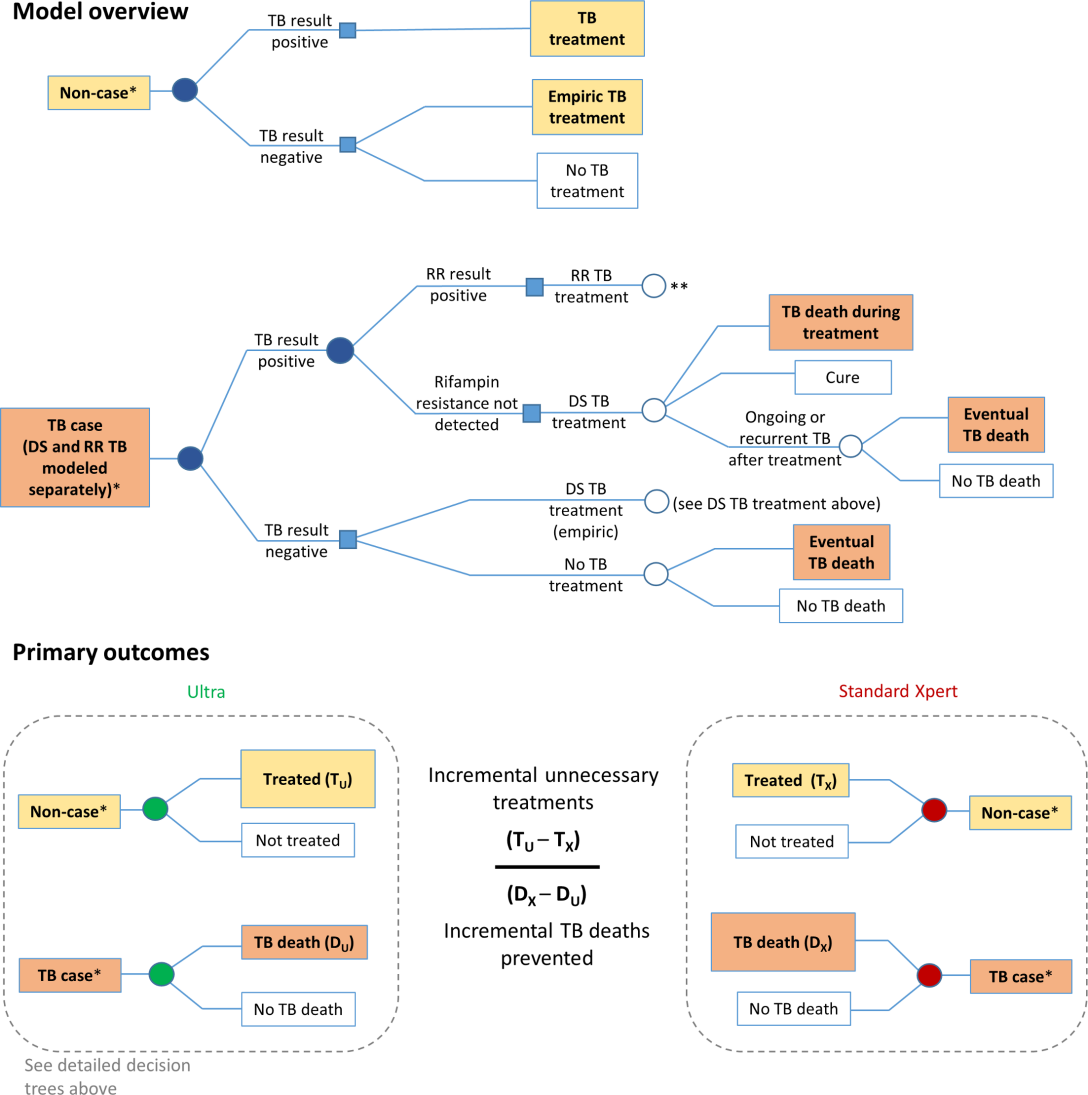
Markov model description. The model diagram shows how an individual suspected of having TB progresses through diagnostic evaluation, treatment decisions, and clinical outcomes. Filled circles indicate diagnostic evaluation with Xpert MTB/RIF (either standard Xpert or Ultra). The lower panels illustrate how primary outcomes—incremental unnecessary TB treatments resulting from Ultra, incremental TB deaths prevented by Ultra, and their ratio—are determined. *Each individual (whether a case or a non-case) is also assigned an HIV status, TB treatment history, age, and sex; these determine the subsequent probabilities within the Markov model. **RR TB treatment is followed by the same decision trees as DS TB treatment, but with different associated probabilities of death and cure, as shown in Table 2. DS, drug-susceptible; RR, rifampin-resistant; TB, tuberculosis.

Within each setting-specific cohort, the values of cohort-defining parameters from Table 1 were used to randomly assign each of 100,000 individual simulated patients an age, sex, underlying TB (and rifampin resistance) status, HIV status, and history of previous TB treatment. Using the additional parameters in Tables 1 and 2, we then simulated individual-level diagnostic evaluation, resulting treatment decisions, and ultimate clinical outcomes for each person in each setting-specific cohort (as illustrated in Fig 1). To accomplish this, we defined a “diagnostic episode” as consisting of all clinical decision-making from the time that a patient is considered at sufficient risk of pulmonary TB to merit Xpert testing to the time that the patient is lost to follow-up, started on TB treatment, or no longer thought to have TB. A diagnostic episode may span multiple visits but, to be included in this analysis, must at some point include a diagnostic evaluation for adult pulmonary TB using either standard Xpert or Ultra. We model the diagnostic episode for each patient in the simulated cohort, assuming that those who test positive with Xpert are initiated on TB treatment (including second-line TB treatment if rifampin resistance is detected). For those who test negative, we assume a setting-specific probability of empiric treatment (i.e., initiating treatment for TB in the absence of a bacteriological result). In order to focus on the clinical impact of Xpert testing, we do not explicitly model other ancillary tests (e.g., chest X-ray, antibiotic trials) but rather assume for simplicity that the results of any such tests performed, coupled with clinical judgement, result in empiric TB treatment for a proportion of Xpert-negative patients. We then vary this empiric treatment proportion directly in sensitivity analysis. We assume that all such empiric treatments involve first-line therapy. Importantly, we also assume outcomes for rifampin-resistant TB that are better than those currently reported, in order not to bias findings against Ultra in light of pharmaceutical and other advances that are likely to improve those outcomes in the future.

**Table 2:** Other model parameters^a^.

Parameter	Category	Value
***Assay-related parameters***		
**Sensitivity for adult pulmonary TB**		
Sensitivity of standard Xpert	HIV−	89.9% (84.2, 94.1)
	HIV+	76.5% (68.0, 84.0)
Sensitivity of Ultra (with trace call^b^) if standard Xpert false-negative	HIV−	18.8% (10.4)^c^
	HIV+	63.0% (9.6)^c^
Sensitivity of Ultra (with trace call^b^) if standard Xpert positive	HIV−	99.3% (0.9)^c^
	HIV+	97.7% (1.9)^c^
Overall sensitivity of Ultra (with trace call), simulated^d^	HIV−	90.9% (86.4, 94.4)
	HIV+	89.7% (83.1, 94.6)
**Specificity for adult pulmonary TB**		
Specificity of standard Xpert	No TB history	98.4% (97.1, 99.1)
	With TB history	98.0% (95.4, 99.3)
Probability of false-positive Ultra (with trace call^b^) if standard Xpert negative	No TB history	2.2% (0.6)^c^
	With TB history	4.9% (1.5)^c^
Probability of false-positive Ultra (with trace call^b^) if standard Xpert false-positive	No TB history	83.3% (11.5)^c^
	With TB history	100%
Overall specificity of Ultra (with trace call), simulated^d^	No TB history	96.3% (94.9, 97.6)
	With TB history	92.9% (89.4, 95.7)
**Sensitivity for rifampin resistance**		
Sensitivity for rifampin resistance, if standard Xpert and Ultra positive for TB (assumed same for both assays)	All cases	95% (91, 98)
Sensitivity for rifampin resistance, if only Ultra positive for TB	All cases	57% (25, 84)
**Specificity for rifampin resistance (assumed same for both assays)**	All cases	98% (96, 99)
***Additional treatment- and outcome-related parameters***		
**Case fatality ratio for RR TB**^**e**^	All cases	43% (25, 65) [10]
**Probability of cure if no death during treatment**		
Rifampin-susceptible TB on first-line treatment^f^	All cases	92% (85, 96) [10]
RR TB on first-line treatment	All cases	15% (0, 30) [15,16]
Any TB on second-line (RR) TB treatment^g^	All cases	85% (80, 90) [17]
**Probability of acquiring RR TB during treatment**	All cases	1% (0.5, 2) [18,19]
**Probability of TB death during treatment**		
Rifampin-susceptible TB on first-line therapy^h^	HIV−	4% (2, 6) [10]
	HIV+	12% (9, 15) [10]
RR TB and/or second-line therapy^g^	HIV−	6% (4, 10) [20]
	HIV+	12% (8, 20) [10,20]

^a^Values shown represent the mode and range of sampled triangular distributions, except where otherwise noted.

^b^Parameter values for scenarios with no trace call and with trace calls repeated are shown in S1 Table.

^c^Values shown are the mean (standard deviation) of sampled beta distributions, chosen to match 95% binomial confidence intervals determined in the clinical study of Ultra [5].

^d^The sensitivity and specificity of Ultra for TB were modeled as conditional on the standard Xpert result (in order to capture the amount of correlation between the 2 assays). The absolute sensitivity and specificity values were calculated for each simulation, and the median (inner 95 percentile range) over all simulations is shown here for reader clarity.

^e^Case fatality for drug-susceptible TB varied considerably by setting and is included in Table 1 of setting-specific parameters.

^f^The cure probability shown for rifampin-susceptible TB is for the Indian TB center and South African HIV clinic settings. In the Chinese primary care setting, a higher data-consistent cure probability of 96% (94%, 98%) was used.

^g^Treatment outcomes (cure and death probabilities) for rifampin-resistant TB are based on expectations of improving treatment outcomes as more effective drugs and diagnostics become available; this estimate is detailed in S1 Supplemental Methods and explored further in a sensitivity analysis.

^h^Values shown for rifampin-susceptible TB are for the Indian setting. Corresponding parameter values for other settings, also based on national TB program data reported by WHO, were 8% (4%, 12%) in the modeled South African setting (an entirely HIV+ cohort), 1% (0%, 3%) for HIV− individuals in China, and 9% (6%, 12%) for HIV+ individuals in China.

RR, rifampin-resistant; TB, tuberculosis; Xpert, Xpert MTB/RIF.

Following the outcome of the diagnostic episode (treated for drug-susceptible or rifampin-resistant TB, or not treated), we then model both treatment outcomes and the ultimate probability of TB death. For those who are treated, treatment outcomes include cure/treatment success, death (due to TB or other causes), and failure/relapse (with the possibility of acquired rifampin resistance), with probabilities based on data reported to the World Health Organization from each country. For individuals with active TB who are not successfully treated (or not treated at all), we do not explicitly model all future clinical care (including possible subsequent diagnostic episodes and/or TB treatment) but rather assume a probability of ultimate TB death equal to the reported case fatality ratio of TB or multidrug-resistant TB in each country, stratified by HIV status. This probability is also varied directly in sensitivity analysis.

### Clinical outcomes

For each simulated cohort, we compared clinical outcomes under 2 alternative scenarios: one with the use of the standard Xpert cartridge and one with the use of the Ultra cartridge. We defined 3 a priori co-primary outcomes, each measured as the expected incremental value if standard Xpert were switched to Ultra: (a) incremental TB-attributable deaths averted, (b) incremental unnecessary TB treatments, and (c) the ratio of these 2 competing outcomes (incremental unnecessary treatments per incremental TB death averted). TB-attributable deaths include all TB-attributable deaths during treatment, after unsuccessful treatment, or after a missed diagnosis. Unnecessary TB treatments include treatments of people without underlying TB, due to false-positive Xpert result or incorrect empiric treatment (assuming that switching from the standard Xpert to the Ultra cartridge does not change the proportion of Xpert-negative patients to whom empiric treatment is prescribed). As a proxy for avertible transmission potential, we also considered as secondary outcomes the difference in the number of TB cases and the number of rifampin-resistant TB cases that remained untreated after the diagnostic attempt using either standard Xpert or Ultra.

### Data inputs

To compare the standard Xpert cartridge against the Ultra cartridge, we assumed accuracy values as shown in Table 2, reflecting data from the recently performed diagnostic accuracy study among adults with symptoms of pulmonary TB at 10 sites in 8 countries, using mycobacterial culture as a reference standard [5]. Basing estimates on study data, the sensitivity and specificity for TB of Ultra were represented as beta distributions conditional on standard Xpert result (i.e., different for those with a positive versus negative standard Xpert result), with mean and standard deviation based on the confidence intervals reported in the trial. The sensitivity of Ultra for TB was also stratified by HIV status, and the specificity for TB was stratified by prior TB history. We also estimated the sensitivity and specificity of each assay for rifampin resistance as shown in Table 2.

The Ultra cartridge also has an additional semi-quantitative category on the lower end of the spectrum (“trace call”) indicating very low levels of mycobacterial DNA amplified. In our primary analysis, we included this trace call as a positive result, per the existing configuration of the test (for maximum sensitivity of Ultra). In a secondary analysis, we considered alternative approaches to interpretation of Ultra in which a trace call was assumed to represent a negative result—either for all individuals or only for those individuals with a prior history of TB treatment (“conditional trace call” scenario). We also considered an approach in which a trace call triggered a repeat Ultra test for adjudication (“positive trace repeated” scenario).

S1 Supplemental Methods provides details of the estimation of other parameters not directly related to the diagnostic assays.

### Uncertainty and sensitivity analysis

For each of the 3 clinical scenarios (100,000 simulated adults each, which for the Indian TB center setting represents annual presumptive TB patients drawn from a general population of approximately 5 million people), we used Latin hypercube sampling to repeatedly draw random sets of all the parameters shown in Table 2 and in the last three rows of Table 1, assuming triangular distributions with the mode and upper/lower bounds provided in the tables (except for the beta distributions used for Ultra sensitivity and specificity as described above). We sampled 5,000 random parameter sets after verifying that this was sufficient to yield consistent results between sets of simulations (S3 Table). Each parameter set was then used to inform a stochastic simulation of diagnostic, treatment, and clinical outcomes, in which we first ran a simulation assuming the use of standard Xpert and then performed a counterfactual simulation differing from the initial simulation only by the replacement of standard Xpert with Ultra. Incremental outcomes were then evaluated by comparing results between the initial and counterfactual simulations; this process was repeated for each of the 5,000 parameter sets, in each clinical scenario. We report 95% uncertainty ranges (URs) as the 2.5th to 97.5th percentile of results from these 5,000 paired (initial and counterfactual) simulations. These URs thus reflect uncertainty in underlying parameter values (over the 5,000 random sets drawn), stochastic process uncertainty (as each of the 5,000 simulations represents a different stochastic realization), and the expected correlation between the results of standard Xpert and Ultra (by evaluating incremental outcomes from paired initial and counterfactual simulations).

We performed 1-way sensitivity analysis on all model parameters across the ranges specified in Tables 1 and 2, using partial rank correlation coefficients to control for potential variation in other model parameters. We then performed 3-way sensitivity analysis across 3 influential setting-specific parameters (TB prevalence, HIV prevalence, and TB-associated mortality rate).

Several additional sensitivity analyses considered alternative estimates for specific sets of parameters. To capture the possibility of lower specificity of Xpert and Ultra in settings of higher TB incidence (observed in a post hoc analysis of diagnostic accuracy study data after adjusting for participants’ personal history of TB, and possibly reflecting a greater probability of previously unrecognized, spontaneously resolved TB or inhalation of nonviable *Mycobacterium tuberculosis*), we ran additional simulations for each setting using specificity estimates based only on data from study sites with correspondingly high or low TB incidence. For the Chinese primary care setting scenario, we repeated simulations with the specificities of standard Xpert and Ultra reestimated after restricting the primary study data to the 4 countries with estimated national TB incidence < 100/100,000 person-years, while for the Indian TB center and South African HIV clinic settings, we repeated simulations with assay specificities reestimated using data only from study sites in countries with national TB incidence ≥ 100/100,000 person-years (S5 Table). This stratification of specificity by national TB incidence corresponds to a post hoc analysis performed on data from the diagnostic accuracy study of Ultra. In another sensitivity analysis performed at the request of a reviewer, we considered worse treatment outcomes for rifampin-resistant TB, consistent with outcomes reported by WHO for 2013 multidrug- or rifampin-resistant TB cohorts: a 14% (10%, 20%) probability of TB mortality during treatment and a 63% (54%, 72%) probability of cure among those who survive treatment [10]. Other sensitivity analyses also performed at the request of reviewers consider empiric treatment in the Chinese primary care setting and changes in clinician behavior (e.g., lower levels of empiric treatment, increased use of confirmatory testing) resulting from their knowledge of Ultra’s lower specificity. Details of the parameter values used in these analyses are provided in S1 Supplemental Methods. Finally, we considered the potential impact of the imperfect sensitivity of TB culture as a “gold standard,” such that some positive Ultra results originally classified as false-positives (i.e., culture-negative) were reclassified as true positives (details in S1 Supplemental Methods).

The model was implemented using R version 3.2.2 [21]. We have made the model code available at https://github.com/eakendall/xpert-ultra.

## Results

### Primary results: TB deaths and unnecessary treatments

The primary outcomes, by clinical setting, are shown in Table 3. In the Indian TB center setting, for example, switching from standard Xpert to Ultra resulted in appropriate treatment for a median of 3% (95% UR: 0.4%, 5.5%) more TB cases (where “appropriate” is defined as second-line treatment for those cases with rifampin resistance and any TB treatment for other TB cases), increasing the median proportion appropriately treated from 88.5% (95% UR: 84.5%, 92.0%) to 91.5% (95% UR: 87.2%, 94.8%). However, switching to Ultra also increased the median proportion of people without TB being unnecessarily treated by 2.1% (95% UR: 1.1%, 3.2%). Since we assumed in this setting that nearly 8 individuals without TB would be evaluated for every case of true TB, Ultra resulted in a median of 5.2 (95% UR: 1.9, 19.1) additional unnecessary TB treatments for every additional TB case detected (S1 Fig). After modeling long-term effects on mortality, use of Ultra rather than standard Xpert was projected to avert 0.5 TB deaths (95% UR: 0, 1.3) and generate 18 unnecessary treatments (95% UR: 10, 29) per 1,000 individuals evaluated—for a median ratio of 38 unnecessary treatments per death averted (95% UR: 12, indefinite upper bound due to simulations in which Ultra averted no deaths). In the HIV care setting in South Africa, where the amount of additional sensitivity added by Ultra is larger and the mortality rates associated with untreated TB are higher, this ratio was more favorable: a median 7 incremental unnecessary treatments per incremental TB death averted (95% UR: 2, 43). By contrast, in the Chinese primary care setting, with lower TB prevalence (15 non-cases evaluated per TB case) and mortality, the median ratio rose to 372 (95% UR: 75, indefinite upper bound) unnecessary treatments per TB death averted.

**Table 3:** Primary outcomes per 1,000 individuals evaluated in 3 clinical settings.

Outcome	Standard Xpert, median (95% UR)	Ultra, median (95% UR)	Difference (or ratio of differences), Ultra versus standard Xpert, median [80% UR] (95% UR)
**TB deaths**			
Indian TB center	10.4 (7.6, 14.3)	9.9 (7.3, 13.4)	−0.5 [−1.0, −0.2] (−1.3, 0.0)
South African HIV clinic	15.4 (10.8, 21.2)	13.9 (9.9, 18.8)	−1.4 [−2.8, −0.6] (−3.7, −0.3)
Chinese primary care	2.1 (1.5, 2.9)	2.1 (1.4, 2.9)	−0.1 [−0.1, 0] (−0.2, 0.1)
**Unnecessary TB treatments**			
Indian TB center	56 (38, 80)	75 (55, 100)	18 [13, 25] (10, 29)
South African HIV clinic	363 (229, 497)	373 (241, 505)	10 [7, 15] (5, 19)
Chinese primary care	17 (10, 25)	35 (24, 49)	18 [22, 26] (8, 30)
**Unnecessary treatments per TB death averted**			
Indian TB center	—	—	38 [17, 125] (12, *)
South African HIV clinic	—	—	7 [3, 19] (2, 43)
Chinese primary care	—	—	372 [118, *] (75, *)

*Upper bound not determined because more deaths occurred with Ultra than with standard Xpert in >2.5% (or for 80% UR, >10%) of simulations.

TB, tuberculosis; UR, uncertainty range; Xpert, Xpert MTB/RIF.

### Missed diagnoses

We also estimated the increase in the number of patients started on appropriate treatment after evaluation with Ultra versus standard Xpert (S6 Table). Per 1,000 individuals evaluated with Xpert in the Indian public TB center, for example, Ultra led to an additional 3.4 (95% UR: 0.7, 6.2) prompt treatment initiations for drug-susceptible TB and an additional 0.04 (95% UR: −0.1, 0.2) prompt second-line treatment initiations for rifampin-resistant TB (S6 Table). The resulting reduction in transmission (before these cases would otherwise be diagnosed) was not estimated in this analysis, but is likely to be small given the paucibacillary nature of those cases detected by Ultra but not detected by standard Xpert.

### Role of the trace call

**Fig 2:**
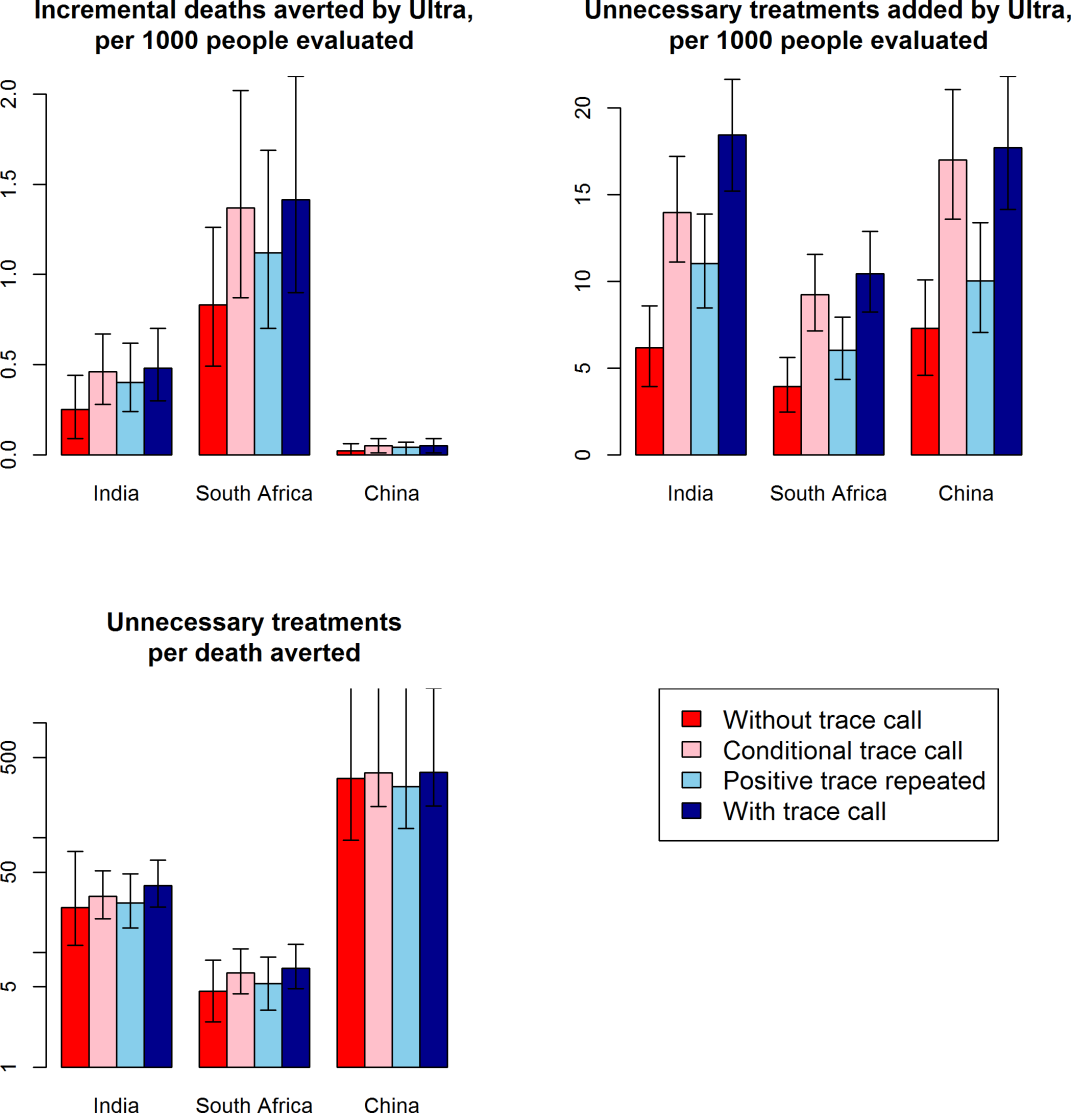
Impact of the Xpert Ultra trace call. Shown are expected primary outcomes under different scenarios for use of the trace call: trace results treated as negative (“without trace call,” red bars), trace results treated as positive only for those with no history of previous TB (“conditional trace call,” light red bars), trace results repeated and treated as positive only if the repeat result is trace or fully positive (“positive trace repeated,” light blue bars), or trace results treated as positive (“with trace call,” the primary analysis, dark blue bars). Bar graphs show the median over 5,000 simulations comparing standard Xpert to Ultra, and error bars show the interquartile range (25th and 75th percentile) of simulations; where no upper error bar is shown, no deaths were prevented in >25% of simulations. Treating the trace call as positive increased both incremental deaths averted and incremental unnecessary treatments but had little impact on the ratio of these 2 outcomes. TB, tuberculosis; Xpert, Xpert MTB/RIF.

Exclusion of the trace call reduced the incremental number of unnecessary treatments (with Ultra versus standard Xpert) by more than 50% in all settings, but also reduced the incremental number of deaths averted by similar proportions (Fig 2; S4 Table). In general, the choice of whether and how to include the result of the trace call resulted in little change in the ratio of additional unnecessary treatments per TB death averted. Differences in outcomes between clinical settings were substantially larger than the differences in outcomes comparing different approaches to the trace call (Fig 2).

### Sensitivity analyses

The estimated number of TB deaths averted was most sensitive to variation in the case fatality ratio for drug-susceptible TB (i.e., the probability of subsequent death following a missed diagnosis of TB) and to the sensitivity of Ultra among individuals with the cohort’s predominant HIV status (S3 Fig). By contrast, the incremental number of unnecessary treatments was highly sensitive to the estimated specificity of Ultra (S3 Fig). Characteristics of the clinical cohorts—which were held fixed for each setting in our primary analysis—were also important. For example, a 2-fold increase in the prevalence of TB (among those with symptoms) or a 4-fold increase in the prevalence of HIV each had similar effects as a 2-fold increase in TB case fatality (S4 Fig).

When specificity parameter estimates for the Indian TB center and the South African HIV clinic were based only on data from study sites in higher-incidence countries (a post hoc analysis of data from the diagnostic accuracy study), the performance of Ultra became somewhat less favorable in those settings, with an increase in the expected number of unnecessary treatments per death prevented from 38 to 55 in the Indian TB center and from 7 to 10 in the South African HIV clinic (Table 4). Conversely, because specificity estimates based only on data from study sites in lower-incidence countries had largely overlapping confidence intervals for standard Xpert and Ultra (S5 Table), the expectation that Ultra would result in more unnecessary treatments became uncertain when using data on specificity only from lower-burden settings; the median number of unnecessary treatments per death averted in the Chinese primary care setting was reduced from 372 to 14 in this sensitivity analysis, but with very large uncertainty, ranging from no added unnecessarily treatments to a ratio of >1,000 (Table 4).

**Table 4:** Sensitivity analysis using specificity estimates from a post hoc analysis of specificity differences in high- versus low-TB burden settings.

Outcome	Standard Xpert^a^	Ultra^a^	Difference (or ratio of differences), Ultra versus standard Xpert^a^
***Results for Indian TB center*, *reestimating specificity using only data from diagnostic accuracy study sites in higher-incidence countries (countries with TB incidence*** *≥* ***100/100*,*000 person-years)***^***b***^			
**TB deaths**			
Original specificity estimate	10.4 (7.6, 14.3)	9.9 (7.3, 13.4)	−0.48 (−1.3, 0.0)
Higher-incidence specificity estimate	10.4 (7.6, 14.3)	9.9 (7.3, 13.4)	−0.48 (−1.3, 0.0)
**Unnecessary TB treatments**			
Original specificity estimate	56 (38, 80)	75 (55, 100)	18 (10, 29)
Higher-incidence specificity estimate	62 (43, 87)	90 (66, 117)	27 (15, 42)
**Unnecessary treatments per TB death averted**			
Original specificity estimate	*—*	*—*	38 (12, *)
Higher-incidence specificity estimate	*—*	*—*	55 (18, *)
***Results for South African HIV clinic*, *reestimating specificity using only data from diagnostic accuracy study sites in higher-incidence countries (countries with TB incidence*** *≥* ***100/100*,*000 person-years)***^***b***^			
**TB deaths**			
Original specificity estimate	15.4 (10.8, 21.2)	13.9 (9.9, 18.8)	−1.42 (−3.7, −0.3)
Higher-incidence specificity estimate	15.4 (10.8, 21.2)	13.9 (9.9, 18.8)	−1.42 (−3.7, −0.3)
**Unnecessary TB treatments**			
Original specificity estimate	363 (229, 497)	373 (241, 505)	10 (5, 19)
Higher-incidence specificity estimate	367 (235, 399)	382 (253, 512)	15 (7, 26)
**Unnecessary treatments per TB death averted**			
Original specificity estimate	*—*	*—*	7 (2, 43)
Higher-incidence specificity estimate	*—*	*—*	10 (3, 61)
***Results for Chinese primary care clinic*, *reestimating specificity using only data from diagnostic accuracy study sites in lower-incidence countries (countries with TB incidence < 100/100*,*000 person-years)***^***b***^			
**TB deaths**			
Original specificity estimate	2.12 (1.5, 2.9)	2.06 (1.4, 2.9)	−0.05 (−0.2, 0.1)
Lower-incidence specificity estimate	2.12 (1.5, 2.9)	2.06 (1.4, 2.9)	−0.05 (−0.2, 0.1)
**Unnecessary TB treatments**			
Original specificity estimate	17 (10, 25)	35 (24, 49)	18 (8, 30)
Lower-incidence specificity estimate	8 (2, 17)	9 (1, 26)	1 (−11, 19)
**Unnecessary treatments per TB death averted**			
Original specificity estimate	*—*	*—*	372 (75, *)
Lower-incidence specificity estimate	*—*	*—*	14 **

^a^Median (95% uncertainty interval).

^b^Reestimated parameter values are shown in S6 Table.

*Upper bound not determined because more deaths and/or fewer unnecessary treatments occurred with Ultra than with standard Xpert in >2.5% of simulations.

**Reported value is the ratio of median estimates for deaths averted and unnecessary treatments. (Median of ratio could not be calculated because Ultra failed to avert deaths in 23% of simulations and also resulted in fewer unnecessary treatments in 46% of simulations.) The associated broad uncertainty range includes possibilities of no mortality benefit with Ultra, of no additional unnecessary treatments with Ultra, and of >1,000 additional unnecessary treatments per death prevented by Ultra.

TB, tuberculosis; Xpert, Xpert MTB/RIF.

Assuming more pessimistic future treatment outcomes for rifampin-resistant TB increased TB deaths in both the standard Xpert scenario and the Ultra scenario but had little impact on the primary results of deaths averted by Ultra or the ratio of unnecessary treatments per death averted (S7 Table). Adding empiric TB treatment in the Chinese primary care setting also had little impact on the results (S8 Table). Following positive Ultra results with a separate confirmatory test could have a mortality benefit only if the confirmatory test were highly sensitive as well as specific; for existing diagnostics, such as chest X-ray, that could be considered as a confirmatory test after screening positive by Ultra, the loss of sensitivity associated with the confirmatory test would outweigh the benefit of the improved specificity (S9 Table). In an HIV care setting with a high rate of empiric treatment following negative results on standard Xpert, a greater confidence in negative Ultra results could substantially reduce unnecessary treatments with a relatively small impact on case detection and TB mortality (S9 Table). Considering the possibility that some culture-negative, Ultra-positive results represented false-negative cultures changed the ratio of unnecessary treatments per death averted by at most a factor of 2 (S10 Table).

## Discussion

Public health decision-making about replacing standard Xpert with Ultra will involve difficult trade-offs. The increased sensitivity of Ultra can lead to lower TB mortality, morbidity, and transmission, but the reduced specificity can result in individuals without TB being unnecessarily exposed to the toxicity and inconvenience of prolonged therapy. A quantitative understanding of these trade-offs—which are likely to be very different in different epidemiologic and clinical contexts—can guide adoption and implementation decisions. We have used a simulation approach to quantify the anticipated clinical consequences of replacing the standard Xpert cartridge with the Ultra cartridge for the diagnosis of adult pulmonary TB in different medium- to high-incidence clinical settings. Depending on the setting and diagnostic algorithm employed, we estimate that use of Ultra for this indication could result in anywhere from fewer than 10 to more than 300 additional unnecessary treatments for every TB death averted. This ratio, which we offer as a tool for understanding the variation in clinical consequences between settings, is most favorable where TB prevalence (especially HIV-associated TB prevalence) and TB mortality are high (e.g., HIV care in South Africa), and, unless preliminary evidence suggesting higher specificity in lower-burden settings is confirmed, it is least favorable where TB prevalence and mortality are lower (e.g., general primary care in China). These findings suggest that the same changes in sensitivity and specificity would have dramatically different consequences in different clinical settings.

We emphasize that the settings we have modeled are intended to represent specific clinical contexts and not all TB diagnostic attempts within a given country. There will be other clinical contexts within these heterogeneous countries (e.g., HIV care settings in India or China) where the relative benefits of standard Xpert versus Ultra will differ from the settings we have modeled in those countries. The practicalities of supply, procurement, and training may make it difficult, however, to offer Ultra alongside standard Xpert for use on a case-by-case basis at the clinic or hospital level or for different diagnostic tasks within the same health system. Rather, decisions to implement Ultra are likely to be made at the level of entire countries and to involve a wide range of clinical settings and potential indications (e.g., active case finding versus symptom-driven diagnosis). The level of overtreatment considered to be acceptable will vary in different social contexts; considerations will include potential strain on healthcare systems (by multiplying the number of people being treated for TB), patient faith in healthcare systems, and preferences regarding the relative harm of under- versus overdiagnosis [22,23]. It is also important to recognize that Ultra may have additional advantages that were not included in this modeling exercise. These include the potential for improved sensitivity (without the same specificity cost) in children [24] and patients with extrapulmonary TB [25]. Although settings with extremely high prevalence of rifampin resistance were not included in the current analysis due to limited data on the relative performance of the 2 Xpert assays for rifampin resistance detection in clinical contexts, small analytical studies demonstrate increased fidelity in rifampin resistance detection and improved specificity in differentiation of non-tuberculous mycobacteria [26], suggesting that Ultra may offer particular benefit in such settings. The decision to implement Ultra may therefore be different in countries with different TB epidemics, different healthcare systems, different societal values, and different relative weightings of the advantages and disadvantages of Ultra.

Importantly, policy decisions about whether and how to implement Ultra may also eventually influence clinical decision-making. For example, confidence among clinicians in the higher sensitivity of Ultra could reduce empiric treatment practices—and the consequences could be either positive (fewer overtreatments) or negative (missed treatment of Ultra false-negatives). In addition, if data suggesting a lower specificity of Ultra are borne out in clinical experience, then the decision to adopt Ultra could, over time, result in more selective use of Xpert tests. Again, as illustrated by our sensitivity analysis regarding such potential changes in practices, this could have positive effects (reducing excessive use of this diagnostic resource) or negative ones (reducing the testing of true cases).

Our analysis is helpful in identifying the key characteristics of settings in which Ultra is likely to be most preferred—namely settings with high prevalence of TB among adult patients likely to be evaluated with Xpert, as well as high prevalence of HIV and high risk of TB mortality if diagnoses are missed. Similarly, we identify characteristics of settings where there is greater risk that the disadvantages of Ultra may outweigh its benefits, and where standard Xpert might be preferred—settings with lower TB prevalence, low HIV prevalence, and low risk of TB mortality. In settings falling between these extremes (such as the illustrative Indian TB center in our model), the choice of cartridge is likely to depend on local priorities, for example, whether it is judged acceptable to subject dozens of people to unnecessary treatment in order to avert 1 death from pulmonary TB. Our analyses also suggest that the clinical setting is likely a much stronger determinant of the risk–benefit ratio in using Ultra than is the use or non-use of the trace call. Within a given setting, use of the trace call appears to increase unnecessary treatments in proportion to the TB deaths it averts. Therefore, in settings where the trade-off between these outcomes is judged to clearly favor Ultra, it is likely to also favor inclusion of trace call diagnoses, whereas disregarding or confirming trace call results may make sense in health systems that adopt Ultra for TB diagnosis but have less confidence that the associated sensitivity gains outweigh the specificity losses in adult pulmonary TB.

The uncertainty in our quantitative estimates remains reasonably high, reflecting in part the challenges of estimating the precise magnitude of sensitivity and specificity differences between Ultra and standard Xpert in multiple types of patients. However, the clinical data to inform these estimates come from a multicenter study of over 1,500 patients, and it is unlikely that additional data on diagnostic performance would greatly improve decision-making. This is because setting-specific parameters (e.g., TB prevalence, TB case fatality) are at least as important as assay-specific parameters. A possible exception in this regard concerns differences in specificity observed in post hoc analysis between study sites with different TB incidence, even among individuals with no history of TB. If, as experience with this assay accumulates, higher specificity in lower-incidence settings continues to be observed, then our results may be pessimistic with respect to use of Ultra in those settings. On the other hand, if specificity in higher-incidence settings is lower than modeled here, use of Xpert Ultra in those settings could be more problematic. To the extent that Ultra test characteristics are consistent between epidemiologic settings, our model’s ability to inform decision-making in any given setting will be primarily limited by our ability to describe the epidemiology of that setting, not by uncertainty regarding the diagnostic accuracy of Ultra. For example, our model suggests that the probability of TB death after a missed diagnosis is a critical parameter value—and this value is poorly understood in most settings [27]. Moreover, clinical and policy decisions are likely to be made on a semi-quantitative basis at best. For example, narrowing the confidence intervals of these quantitative estimates is less likely to influence decision-making than is a qualitative assessment of whether the loss of specificity associated with Ultra is acceptably small or unacceptably large, compared to the deaths averted and other potential benefits.

This analysis has a number of important limitations. We did not model the transmission of TB and thus may have underestimated the impact of Ultra after accounting for secondary transmission from cases diagnosed by Ultra but not standard Xpert. However, the amount of transmission from Xpert-negative TB cases is uncertain and likely to be low in settings where these cases would eventually come to clinical attention [28–30]. We also did not account for long-lasting sequelae of TB disease and delayed diagnosis [31], focusing instead on TB mortality (which represents the vast majority of disability-adjusted life years in other studies of TB disease [32–34]). We restricted our analysis to evaluation of adults presenting with symptoms of pulmonary TB in medium- to high-TB-burden settings. For other potential uses (e.g., diagnosis of extrapulmonary [35] or pediatric [36] TB, and use in low-prevalence settings), preliminary data suggest that sensitivity increases may be substantial and may come with less specificity cost [24,25]. For these indications, additional analyses in these specific populations would be warranted once confirmed data are available. Our primary analysis uses pooled data from all diagnostic accuracy study sites (for assay characteristics that were expected to be consistent across sites); if preliminary suggestions of higher specificity in lower-prevalence sites are confirmed, differences in outcomes across settings would be attenuated. Finally, our model does not include data on costs or the implications of false-positive or false-negative results on health utility. Future context-specific health technology assessments would therefore be useful to convert these results into estimates of cost-effectiveness and budget impact across different settings for use in national-level decision-making.

In summary, this individual-based cohort model in 3 illustrative clinical settings demonstrates the clinical implications of the sensitivity/specificity trade-off when replacing standard Xpert with Xpert Ultra for diagnosis of adult pulmonary TB. We demonstrate that this replacement will likely prevent a substantial number of TB deaths in settings characterized by high TB and HIV prevalence and mortality. While less certain, our findings also suggest that switching to Xpert Ultra may result in substantial overtreatment in settings with moderate prevalence of TB and lower mortality risk. To optimize the use of Xpert Ultra to improve TB diagnosis in moderate- and high-burden settings, we must carefully consider the diversity of contexts into which it might be introduced and the complexity of policy recommendations that might ensue.

## Supporting information

S1 Fig Decision trees showing median values for key results in each setting. Results obtained using standard Xpert are shown in red, and results using Ultra are in green. Not shown here but also included in the model are rifampin-resistance status and detection, differential assay and treatment outcomes by HIV status and treatment history, and rare non-TB-related deaths during TB treatment.
(TIF)

S2 Fig Visual representation of setting-specific cohorts and primary outcomes, for the 3 modeled settings (public TB center in India, HIV clinic in South Africa, and general primary care setting in China). The setting-specific cohorts are displayed as stacked bar graphs, then the primary outcomes are highlighted, and finally the stacked bar graphs are rearranged to show the relative magnitudes of the outcomes more clearly and magnified to make visible the small numbers of incremental TB deaths averted by Ultra in the Indian TB center and Chinese primary care settings.
(PNG)

S3 Fig Adjusted 1-way sensitivity analysis for sampled parameter values. Partial rank correlation coefficients (PRCCs) were calculated to determine the sensitivity of each of the 3 primary outcomes, in each of the 3 modeled settings, to the value of each assay- and outcome-related parameter from Tables 1 and 2, after adjusting for all other such parameters. The parameters with the largest-magnitude PRCCs are shown. Empiric treatment probability does not appear for the Chinese primary care setting because we assumed that empiric treatment would not be widely given in a setting with low TB prevalence (though this assumption is relaxed in S8 Table). The clinical cohorts in each setting were held fixed in the primary analysis, but analysis of sensitivity to the characteristics of each cohort is shown in S4 Fig.
(JPG)

S4 Fig Three-way sensitivity analysis of the primary outcome unnecessary treatments per deaths averted to influential characteristics of the clinical cohort. In the left panel, all other parameters are held fixed at their modal values for the Indian TB center setting, except for the case fatality ratio of drug-susceptible (DS) TB (the probability that a TB case that has not yet been diagnosed and treated will ultimately die of TB) and the prevalence of TB within the evaluated cohort; these two parameters are varied on the *y*-axis and *x*-axis, respectively. In the right panel, the prevalence of HIV within the cohort is increased to 20%, with all other parameters taking the same values as in the left panel. Each box on the grid represents 1 pair of simulations (comparing standard Xpert to Ultra) of a cohort of 100,000 individuals evaluated for TB. Uneven gradients reflect stochastic variation between repeated simulations, despite holding all other parameters constant. White squares represent simulations in which no deaths are averted, or the ratio exceeds 1,000 unnecessary treatments per death.
(TIF)

S5 Fig Variability between simulations. The heatmap shows the variation in deaths averted as only 2 of the most influential model parameters vary. Scatter plots show the additional variability in the simulation outcome of TB deaths averted, beyond that shown in the corresponding cross-sections of the heatmap, due to variation in the other model parameters. To generate the scatter plots, one parameter is held fixed at its modal value (as indicated by a dotted line on the heatmap), while the other parameter from the heatmap (shown on the *x-*axis of the scatter plot), as well as all other assay- and outcome-related model parameters in Table 2, are allowed to vary between simulations as in the primary analysis. Each red point in the lower 2 panels represents 1 of the 5,000 simulations performed, with a 95% loess smoother.
(TIF)

S1 Supplemental Methods (DOCX)

S1 Table Alternative parameter values for Ultra’s sensitivity and specificity for TB, with alternative uses of trace call results. (DOCX)

S2 Table Setting-specific cohorts by case status, rifampin susceptibility, and treatment history. (DOCX)

S3 Table Monte Carlo variability across 40 runs of the Markov model. Each run consists of 5,000 separate simulations of 100,000 individuals evaluated for TB.
(DOCX)

S4 Table Primary results with different uses of trace calls, for all modeled settings. The same results are shown graphically in Fig 2.
(DOCX)

S5 Table Reduction in untreated rifampin-susceptible and rifampin-resistant TB cases when using Ultra. (DOCX)

S6 Table Reestimated assay specificities, stratifying study sites by national TB incidence. (DOCX)

S7 Table Results with more pessimistic estimates of future rifampin-resistant TB treatment outcomes. (DOCX)

S8 Table Results for Chinese primary care setting with and without empiric treatment. (DOCX)

S9 Table Results with altered clinical decision-making based on knowledge of Ultra’s different test characteristics. (DOCX)

S10 Table Results in hypothetical scenario of an imperfect reference standard as the cause of some positive Ultra results. (DOCX)

## Abbreviations

TB: tuberculosis
UR: uncertainty range
Xpert: Xpert MTB/RIF
