# Supplementary figures and images for: Estimated clinical impact of the Xpert MTB/RIF Ultra cartridge for diagnosis of pulmonary tuberculosis: A modeling study
Source: PLoS Med. 2017 Dec 14;14(12):e1002472. doi: 10.1371/journal.pmed.1002472 (PMC5730108; doi:10.1371/journal.pmed.1002472)

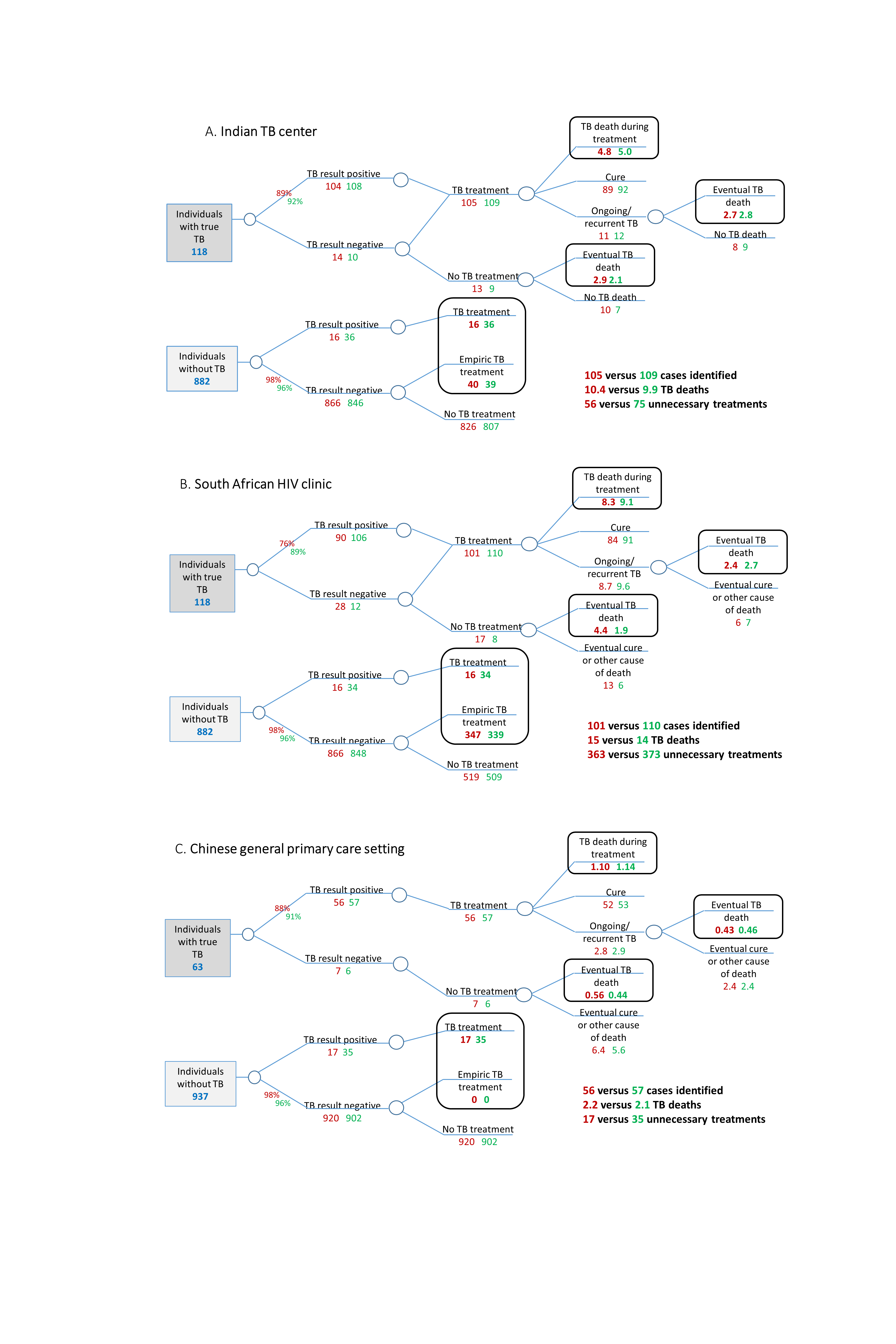

Supplement: S1 Fig — Results obtained using standard Xpert are shown in red, and results using Ultra are in green. Not shown here but also included in the model are rifampin-resistance status and detection, differential assay and treatment outcomes by HIV status and treatment history, and rare non-TB-related deaths during TB treatment. (TIF) [file pmed.1002472.s001.tif]

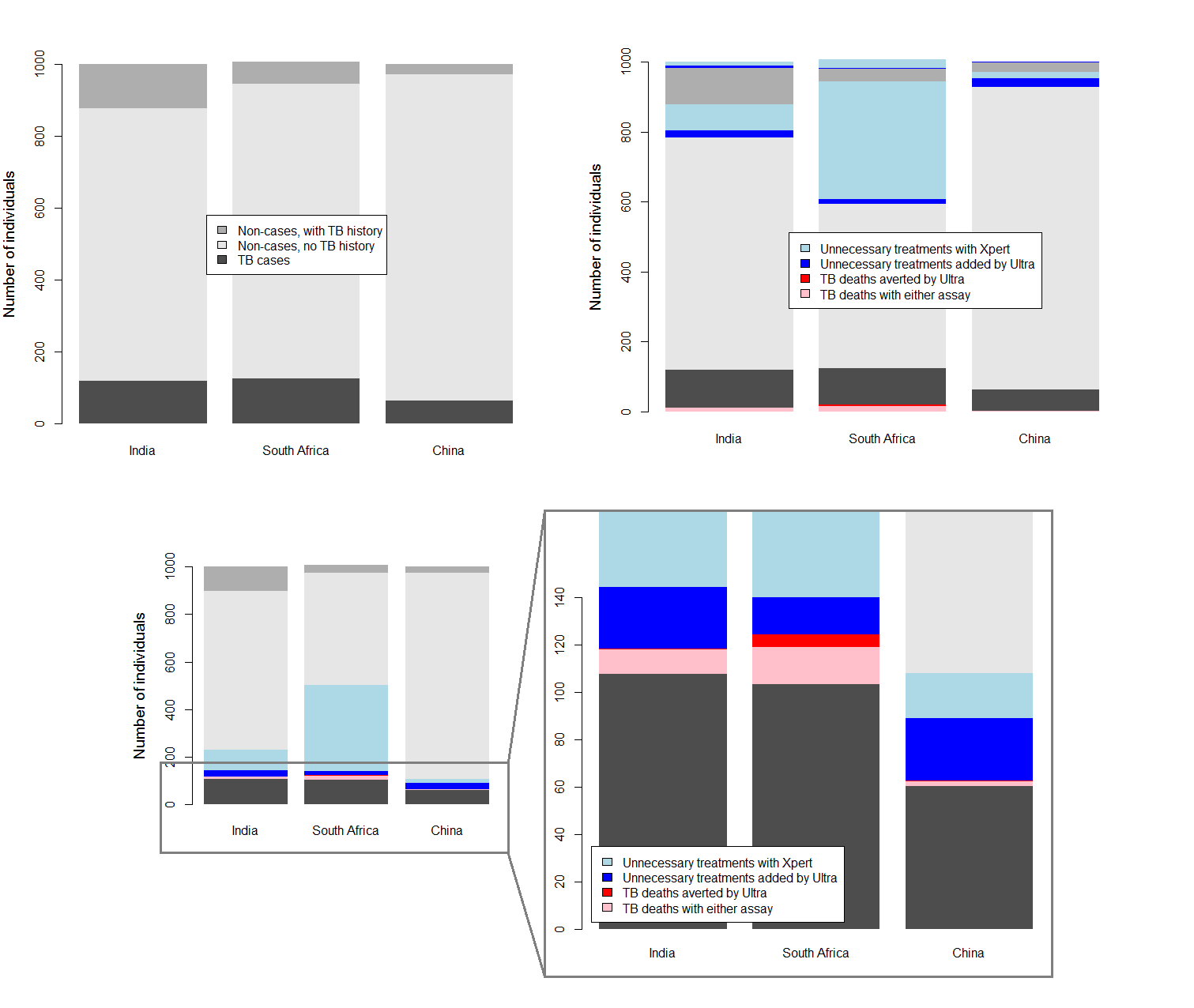

Supplement: S2 Fig — The setting-specific cohorts are displayed as stacked bar graphs, then the primary outcomes are highlighted, and finally the stacked bar graphs are rearranged to show the relative magnitudes of the outcomes more clearly and magnified to make visible the small numbers of incremental TB deaths averted by Ultra in the Indian TB center and Chinese primary care settings. (PNG) [file pmed.1002472.s002.png]

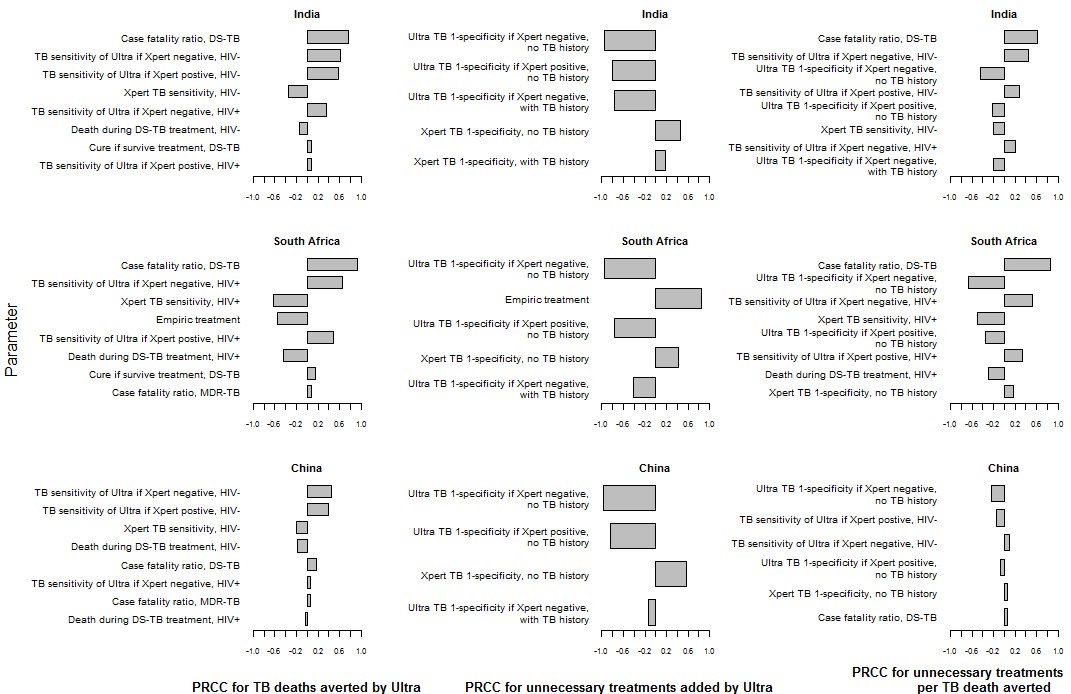

Supplement: S3 Fig — Partial rank correlation coefficients (PRCCs) were calculated to determine the sensitivity of each of the 3 primary outcomes, in each of the 3 modeled settings, to the value of each assay- and outcome-related parameter from Tables 1 and 2, after adjusting for all other such parameters. The parameters with the largest-magnitude PRCCs are shown. Empiric treatment probability does not appear for the Chinese primary care setting because we assumed that empiric treatment would not be widely given in a setting with low TB prevalence (though this assumption is relaxed in S8 Table). The clinical cohorts in each setting were held fixed in the primary analysis, but analysis of sensitivity to the characteristics of each cohort is shown in S4 Fig. (JPG) [file pmed.1002472.s003.jpg]

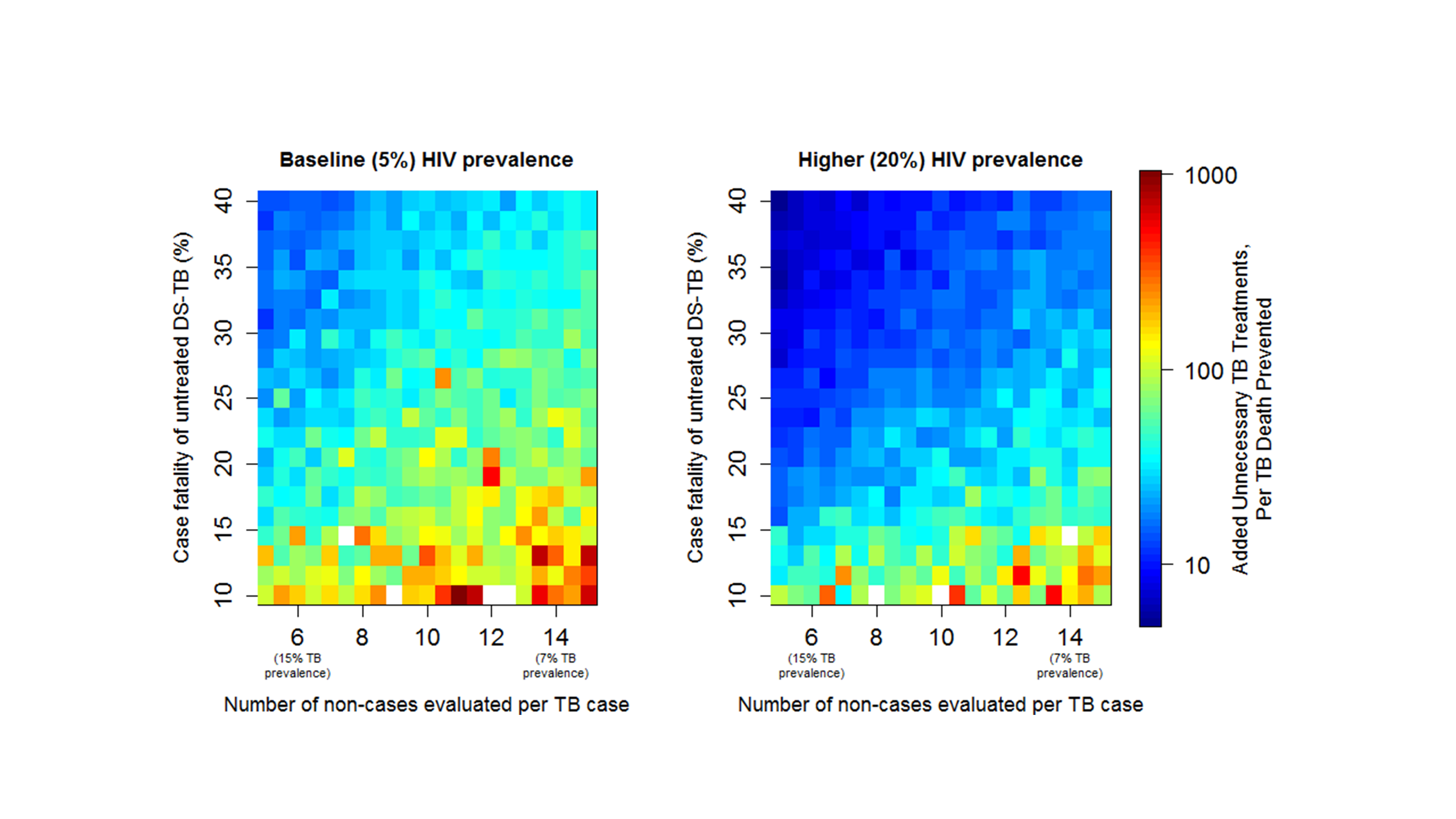

Supplement: S4 Fig — In the left panel, all other parameters are held fixed at their modal values for the Indian TB center setting, except for the case fatality ratio of drug-susceptible (DS) TB (the probability that a TB case that has not yet been diagnosed and treated will ultimately die of TB) and the prevalence of TB within the evaluated cohort; these two parameters are varied on the y-axis and x-axis, respectively. In the right panel, the prevalence of HIV within the cohort is increased to 20%, with all other parameters taking the same values as in the left panel. Each box on the grid represents 1 pair of simulations (comparing standard Xpert to Ultra) of a cohort of 100,000 individuals evaluated for TB. Uneven gradients reflect stochastic variation between repeated simulations, despite holding all other parameters constant. White squares represent simulations in which no deaths are averted, or the ratio exceeds 1,000 unnecessary treatments per death. (TIF) [file pmed.1002472.s004.tif]

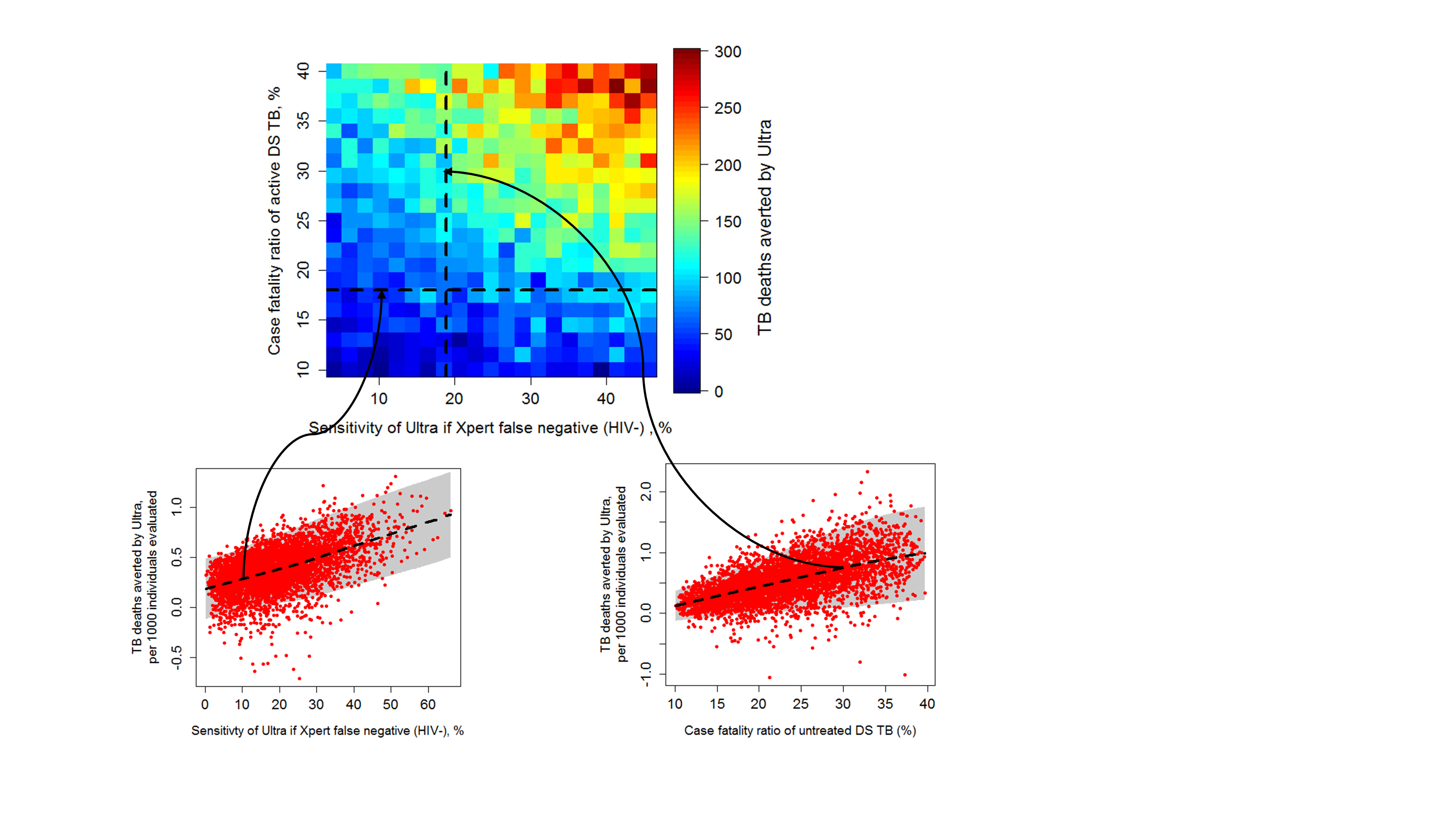

Supplement: S5 Fig — The heatmap shows the variation in deaths averted as only 2 of the most influential model parameters vary. Scatter plots show the additional variability in the simulation outcome of TB deaths averted, beyond that shown in the corresponding cross-sections of the heatmap, due to variation in the other model parameters. To generate the scatter plots, one parameter is held fixed at its modal value (as indicated by a dotted line on the heatmap), while the other parameter from the heatmap (shown on the x-axis of the scatter plot), as well as all other assay- and outcome-related model parameters in Table 2, are allowed to vary between simulations as in the primary analysis. Each red point in the lower 2 panels represents 1 of the 5,000 simulations performed, with a 95% loess smoother. (TIF) [file pmed.1002472.s005.tif]
