# Supplementary material for: Estimated clinical impact of the Xpert MTB/RIF Ultra cartridge for diagnosis of pulmonary tuberculosis: A modeling study
Source: PLoS Med. 2017 Dec 14;14(12):e1002472. doi: 10.1371/journal.pmed.1002472 (PMC5730108; doi:10.1371/journal.pmed.1002472)
Supplement: S1 Supplemental Methods — (DOCX) [file pmed.1002472.s006.docx]

**S1 Supplemental Methods**

Kendall et al., Estimated clinical impact of the Xpert MTB/RIF Ultra cartridge for diagnosis of pulmonary tuberculosis: a modelling study

Derivation of select parameter estimates

*Cohort-related parameters:*

The prevalence of TB among the cohort evaluated for TB in South Africa was based on national laboratory data showing that 1 in 10.4 Xpert assays were positive for TB [1], and assuming 80% average sensitivity of Xpert in this population. The same prevalence was assumed for India; it is also consistent with the TB prevalence among Indian patients with negative or unknown HIV status in Boehme et al 2011 [2] and with the prevalence of up-front Xpert positivity in the implementation study by Sachdeva et al [3]. Similar data are not available for China, but a 50% lower prevalence was selected as both reasonable and to illustrate the impact of Xpert Ultra in a lower-prevalence patient population.

Among cases, the prevalence of rifampin resistance and of prior TB history were assumed to be consistent with national TB notifications [4]; although retreatment cases with rifampin resistance may decrease as rifampin resistance is more effectively identified and treated in new patients, our model assumed the levels reported in 2015.

Few data are available regarding the prevalence of TB history among non-cases evaluated for TB. This prevalence is expected to be lower than that among cases, but higher than that in the general population because TB history and persistent TB risk factors make clinicians more likely to suspect and test for TB. Therefore, we modeled this prevalence as the midpoint between two values: the prevalence in cases, and an estimated prevalence in the overall national population. This overall national prevalence was estimated as the product of the incidence of new TB, the probability that an incident TB case survives (1 - case fatality ratio), and the average expected survival after cure.

In WHO’s TB notification data, age is binned by decade between ages 15 and 65, and in a single bin for those age >65, for each sex. We assigned individual age and sex within each cohort by first sampling from these bins based on the number of notifications in each bin. We then sampled from uniform distributions across each age bin for those age <65, and for those age >65, we sampled from the age distribution reported for those >65 years old in the country’s overall population in the Global Health Observatory data repository [5]. Within each cohort, we used the same distributions of age, sex, and HIV status for non-cases as for cases.

*Treatment- and outcome-related parameters:*

Probabilities of cure, death, or ongoing TB were estimated using data reported by WHO. Importantly, model parameters are not intended to directly map to programmatic outcomes, but rather reflect true clinical status following treatment. Therefore, for example, parameter values must reflect whether those who “complete treatment,” are “lost to follow up,” or have no reported outcome associated with their notification (a situation particularly associated with the increasing number of notifications from India’s private sector) are cured, die during their initial planned course of treatment, or continue on with TB (e.g. with relapsed disease).

To estimate a probability of death during treatment, for drug-susceptible TB, we started with the total reported fraction of patients dying during treatment according to WHO’s 2016 Global TB Report [6] for a given country and HIV infection status. From this, we subtracted the average background mortality rate times average treatment duration, to yield a probability of death due to TB during treatment.

Probabilities of cure among those who did not die during treatment, for drug-susceptible TB on first-line therapy, were also based on outcomes reported by WHO [6]. We assumed that all treatment successes were truly cured, and conversely we assumed that none of the patients reported as lost to follow up were cured by the current treatment course. We excluded those with unknown outcomes, as well as deaths during treatment due to TB or another cause, from the calculation of this proportion cured.

For rifampin-resistant TB, to reflect the evolving standard of care and advances in diagnostics and therapeutics (including earlier case detection, the potential for patient patients to receive a 9-month regimen, and the increasing availability of second-line drug susceptibility testing and of new drug classes for patients with additional resistance), we assumed that treatment outcomes achieved after a health system chose between standard Xpert and Ultra would be superior to those historically reported for rifampin-resistant TB. We used observational data for the short-course MDR-TB regimen in Bangladesh [7] as a basis for our estimates for the probabilities of death during treatment and of cure, for HIV-negative patients. Although that study’s patient population included some fluoroquinolone-resistant patients, we reduced the reported cure rate by an additional 5% to account for additional late relapses and/or for a higher prevalence of patients with more extensive second-line drug resistance. We assumed that TB mortality during treatment would increase for HIV-positive patients by a similar amount for MDR-TB as for drug-susceptible TB.

The probability that rifampin-resistant TB could be cured by first-line therapy were based on observational studies conducted in historical situations where MDR-TB treatment was not widely available [8,9].

We also modeled a possibility of death from another cause during treatment, based on age-, sex-, and HIV-specific mortality rates as detailed below.

Patients who were not diagnosed or treated in the current diagnostic episode were subsequently assumed to have a probability of eventual TB death equal to overall case fatality ratio of TB (in that country, for drug-susceptible TB, or as reported for MTB-TB globally, for those with rifampin resistance). This could be an underestimate, in so far as these individuals’ disease had advanced enough to bring them to clinical attention and yet had not been treated, but it may also be an overestimate of their mortality given that the individuals whose diagnostic testing was falsely negative are individuals who have demonstrated a propensity to access care and have relatively paucibacillary disease. Therefore, we sampled from a wide uncertainty range around this estimate.

For patients who were unsuccessfully treated, the TB case fatality ratio was similarly used to estimate their subsequent probability of TB mortality; for those who acquired rifampin resistance during their attempted treatment, the MDR-TB case fatality ratio was used.

*Background mortality rates*

For HIV-uninfected individuals in India and China, we used Global Health Observatory data [5] to estimate age- and sex-specific mortality rates as for the general population. For HIV-infected individuals, we used HIV-specific life expectancy data from South Africa [6] and estimated the patients in our cohort, who were in care and being evaluated for TB, as otherwise having the life expectancy of an HIV-infected individual in his/her second year on antiretroviral therapy.

Assumptions for sensitivity analysis: Changes in clinical decision-making after switch to Ultra

We explored scenarios in which clinicians make different treatment decisions based on the lower specificity and higher sensitivity of Ultra versus standard Xpert. Outcomes of Ultra versus standard Xpert, after accounting for these potential changes in clinical decision-making, are shown in S9 Table. Here, we describe the corresponding methodology and assumptions.

We first supposed that, in response to Ultra’s lower specificity, clinicians would follow positive Ultra results with a second confirmatory test of a different type – with patients only treated if both Ultra and confirmatory test were positive. This combined algorithm would improve specificity but reduce sensitivity compared to using Ultra alone. In particular, we considered the use of chest X-ray (CXR) as such a confirmatory test, basing revised parameter estimates on published data on the diagnostic accuracy of CXR in presumptive TB cases:

- Sensitivity of CXR: The frequency of normal CXR in sputum culture-positive TB cases has been reported to range from 4.8% [7] to >10% [8] in HIV-uninfected individuals with pulmonary TB, and from 9% [9] to 22% [10] in HIV-associated pulmonary TB. We therefore estimated that 5% of HIV-negative and 10% of HIV-positive individuals with a true-positive Ultra result would go untreated if CXR confirmation of a positive Ultra result was required.
- Specificity of CXR: In order to achieve the above levels of sensitivity for pulmonary TB detection, studies of receiver operating curves of chest x-ray in suspected TB suggest that a specificity of chest x-ray of approximately 50% would have to be accepted [11,12]. We therefore estimated that 50% of individuals with false-positive Ultra results would avoid unnecessary treatment due to this second step.

In addition to possible changes to the interpretation of positive results, we also modeled possible changes to the interpretation of negative results – namely, by supposing that in response to Ultra’s higher sensitivity, clinicians in a high-HIV-prevalence setting would reduce their use of empiric TB treatment after negative Ultra results. Sensitivity in HIV-infected individuals increases from 77% with standard Xpert to 90% with Ultra, and the two tests’ negative likelihood ratios differ by approximately a factor of 2. We are unaware of any data quantifying clinicians’ level of suspicion for TB in those patients whom they treat empirically, so we assumed that 20% of TB-negative patients with TB symptoms who would be empirically treated after a negative Xpert have clinically-assessed prior odds of TB within a factor or 2 of the clinician’s threshold for empiric treatment, such that a negative Ultra result would lead to a decision not to treat, whereas a negative standard Xpert result would still result in a decision to treat empirically.

Assumptions for sensitivity analysis: Possibility of an imperfect reference standard

We also considered a scenario in which culture is an imperfect reference standard and some Ultra-positive, culture-negative individuals are true TB cases. The primary diagnostic accuracy study of Ultra used multiple sputum cultures to enhance sensitivity, and we assumed 95% combined sensitivity of these multiple cultures (e.g. due to overly-aggressive decontamination of sputum), to represent a lower bound of the sensitivity of this multiple-culture evaluation. We also assumed an ability of Ultra to detect half of these culture-negative TB cases. As shown in S10 Table, this would reduce the ratio of unnecessary treatments to deaths averted by approximately a factor of two in each setting.

Due to the lower overall sensitivity of Ultra compared to culture, the consistency of results on multiple sputum specimens in the primary diagnostic study of Ultra, and a lack of TB cases identified in clinical follow-up of culture-negative/Ultra-positive cases, the number of culture-negative/Ultra-positive individuals who are true TB cases is likely smaller than that modeled in this sensitivity analysis [13].

1. South Africa National Health Laboratory Service. GeneXpert MTB/RIF Progress Report [Internet]. 2017 Mar. Available: http://www.nhls.ac.za/assets/files/GeneXpert%20National%20Report%20March%202017.pdf

2. Boehme CC, Nicol MP, Nabeta P, Michael JS, Gotuzzo E, Tahirli R, et al. Feasibility, diagnostic accuracy, and effectiveness of decentralised use of the Xpert MTB/RIF test for diagnosis of tuberculosis and multidrug resistance: a multicentre implementation study. Lancet Lond Engl. 2011;377: 1495–1505. doi:10.1016/S0140-6736(11)60438-8

3. Sachdeva KS, Raizada N, Sreenivas A, Hoog AH van’t, Hof S van den, Dewan PK, et al. Use of Xpert MTB/RIF in Decentralized Public Health Settings and Its Effect on Pulmonary TB and DR-TB Case Finding in India. PLOS ONE. 2015;10: e0126065. doi:10.1371/journal.pone.0126065

4. World Health Organization. Global Tuberculosis Database [Internet]. Available: http://www.who.int/tb/country/data/download/en/

5. Global Health Observatory data repository [Internet]. World Health Organization; Available: http://apps.who.int/gho/data/?theme=main&vid=61830

6. Johnson LF, Mossong J, Dorrington RE, Schomaker M, Hoffmann CJ, Keiser O, et al. Life expectancies of South African adults starting antiretroviral treatment: collaborative analysis of cohort studies. PLoS Med. 2013;10: e1001418. doi:10.1371/journal.pmed.1001418

7. Ackley SF, Liu F, Porco TC, Pepperell CS. Modeling historical tuberculosis epidemics among Canadian First Nations: effects of malnutrition and genetic variation. PeerJ. 2015;3: e1237. doi:10.7717/peerj.1237

8. Stott SM, Soans B, Bui JQH. Comparison of roentgenographic manifestations of active pulmonary tuberculosis between migrant and non-migrant populations in the Hunter Region. J Med Imaging Radiat Oncol. 2015;59: 668–672. doi:10.1111/1754-9485.12351

9. Aderaye G, Bruchfeld J, Assefa G, Feleke D, Källenius G, Baat M, et al. The relationship between disease pattern and disease burden by chest radiography, M. tuberculosis Load, and HIV status in patients with pulmonary tuberculosis in Addis Ababa. Infection. 2004;32: 333–338. doi:10.1007/s15010-004-3089-x

10. Pepper T, Joseph P, Mwenya C, McKee G-S, Haushalter A, Carter A, et al. Normal chest radiography in pulmonary tuberculosis: implications for obtaining respiratory specimen cultures. Int J Tuberc Lung Dis Off J Int Union Tuberc Lung Dis. 2008;12: 397–403.

11. Breuninger M, Ginneken B van, Philipsen RHHM, Mhimbira F, Hella JJ, Lwilla F, et al. Diagnostic Accuracy of Computer-Aided Detection of Pulmonary Tuberculosis in Chest Radiographs: A Validation Study from Sub-Saharan Africa. PLOS ONE. 2014;9: e106381. doi:10.1371/journal.pone.0106381

12. Rahman MT, Codlin AJ, Rahman MM, Nahar A, Reja M, Islam T, et al. An evaluation of automated chest radiography reading software for tuberculosis screening among public- and private-sector patients. Eur Respir J. 2017;49: 1602159. doi:10.1183/13993003.02159-2016

13. Dorman SE, Schumacher SG, Alland D, Nabeta P, Armstrong DT, King B, et al. Xpert MTB/RIF Ultra for detection of Mycobacterium tuberculosis and rifampin resistance: a multicentre diagnostic accuracy study. Lancet Infect Dis [Internet]. 2017 Nov 30 [cited 2017 Dec 1]. Available from: http://dx.doi.org/10.1016/S1473-3099(17)30691-6.
