# Supplementary material for: Estimated clinical impact of the Xpert MTB/RIF Ultra cartridge for diagnosis of pulmonary tuberculosis: A modeling study
Source: PLoS Med. 2017 Dec 14;14(12):e1002472. doi: 10.1371/journal.pmed.1002472 (PMC5730108; doi:10.1371/journal.pmed.1002472)
Supplement: S1 Table — (DOCX) [file pmed.1002472.s007.docx]

**S1 Table: Alternative parameter values for Ultra’s sensitivity and specificity for TB, with alternative uses of trace call result^a^**

| Sensitivity for TB | HIV- | HIV+ |
| --- | --- | --- |
| *Ultra without trace call:* |  |  |
| Sensitivity if standard Xpert falsely negative | 18.8% (10.4) ^b^ | 40.7% (9.7) ^b^ |
| Sensitivity if standard Xpert positive | 98.6% (1.2) ^b^ | 97.7% (1.9) ^b^ |
| Overall sensitivity, simulated^c^ | 90.3% (85.5, 94.1) | 84.3% (77.0, 90.0) |
| *Ultra with positive trace calls repeated^d^* |  |  |
| Sensitivity if standard Xpert falsely negative | 18.8% (10.4) ^b^ | 52.9% (9.9) ^b^ |
| Sensitivity if standard Xpert positive | 99.3% (1.0) ^b^ | 97.7% (1.9) ^b^ |
| Overall sensitivity, simulated^c^ | 90.9% (86.3, 94.6) | 87.3% (80.4, 92.5) |
| **Specificity for TB** | **No TB history** | **With TB history** |
| *Ultra without trace call:* |  |  |
| Probability of false positive if standard Xpert negative | 1.1% (0.4) ^b^ | 0.8% (0.7) ^b^ |
| Probability of false positive if standard Xpert falsely positive | 83.3% (11.6) ^b^ | 80.0% (15) ^b^ |
| Overall specificity, simulated^c^ | 97.4% (96.2, 98.4) | 97.4% (95.0, 98.9) |
| *Ultra with positive trace calls repeated^d^* |  |  |
| Probability of false positive if standard Xpert negative | 1.4% (0.5) ^b^ | 3.7% (1.3) ^b^ |
| Probability of false positive if standard Xpert falsely positive | 83.3% (11.6) ^b^ | 80.0% (15) ^b^ |
| Overall specificity, simulated^c^ | 97.2% (95.8, 98.2) | 94.6% (91.2, 97.0) |

^a^In an additional alternative algorithm, the trace call result is used only for individuals without a history of TB. (The parameter values are therefore equal to those using the trace call for individuals with no history of TB, and those without the trace call for individuals with prior TB.)

^b^ Values shown are the mean and standard deviation of sampled beta distributions, chosen to match 95% binomial confidence intervals determined in the clinical study of Ultra.

^c^ Sensitivities and specificities of Ultra for TB were modeled as conditional on the standard Xpert result (in order to capture the amount of correlation between the two assays), but the absolute sensitivity and specificity values were calculated for each simulation, and the median (inner 95 percentile range) over all simulations are shown.

^d^Ultra repeated if initial result positive by trace call; considered positive only if repeat result is positive with or without trace call.
