# Supplementary material for: Estimated clinical impact of the Xpert MTB/RIF Ultra cartridge for diagnosis of pulmonary tuberculosis: A modeling study
Source: PLoS Med. 2017 Dec 14;14(12):e1002472. doi: 10.1371/journal.pmed.1002472 (PMC5730108; doi:10.1371/journal.pmed.1002472)
Supplement: S2 Table — (DOCX) [file pmed.1002472.s008.docx]

**S2 Table: Setting-specific cohorts by case status, rifampin susceptibility, and treatment history**

|  | **India** | **South Africa (HIV+ only)** | **China** |
| --- | --- | --- | --- |
| **Drug-susceptible TB, HIV-** | 10.6% | 0% | 5.6% |
| **Rifampin-resistant TB, HIV-** | 0.6% | 0% | 0.5% |
| **Drug-susceptible TB, HIV+** | 0.6% | 11.3% | 0.2% |
| **Rifampin-resistant TB, HIV+** | 0.03% | 0.5% | 0.01% |
| **Non-TB, no TB history** | 75.9% | 82.1% | 90.8% |
| **Non-TB, with TB history** | 12.4% | 6.2% | 2.9% |
| **Total** | 100% | 100% | 100% |
