# Supplementary material for: Estimated clinical impact of the Xpert MTB/RIF Ultra cartridge for diagnosis of pulmonary tuberculosis: A modeling study
Source: PLoS Med. 2017 Dec 14;14(12):e1002472. doi: 10.1371/journal.pmed.1002472 (PMC5730108; doi:10.1371/journal.pmed.1002472)
Supplement: S3 Table — Each run consists of 5,000 separate simulations of 100,000 individuals evaluated for TB. (DOCX) [file pmed.1002472.s009.docx]

**S3 Table: Monte Carlo variability across 40 runs of the Markov model, where each run consists of 5000 separate simulations of 100,000 individuals evaluated for TB**

|  | Indian TB clinic | South African HIV clinic | Chinese primary care clinic |
| --- | --- | --- | --- |
| Difference in TB deaths, Ultra vs standard Xpert |  |  |  |
| Values reported in manuscript | -0.48 (-1.3, 0.0) | -1.42 (-3.7, -0.3) | -0.05 (-0.2, 0.1) |
| Median (95% range) of reported median | -0.48 (-0.47, -0.49) | -1.41 (-1.39, -1.43) | -0.05 (-0.05, -0.05) |
| Median (95% range) of reported lower bound of 95% UR | -1.26 (-1.23, -1.29) | -3.67 (-3.59, -3.75) | -0.19 (-0.19, -0.18) |
| Median (95% range) of reported upper bound of 95% UR | 0.03 (0.01, 0.05) | -0.26 (-0.22, -0.28) | 0.08 (0.07, 0.09) |
| Difference in unnecessary TB treatments, Ultra vs standard Xpert |  |  |  |
| Values (median (95% UR)) reported in manuscript | 18 (10,29) | 10 (5, 19) | 18 (8, 30) |
| Median (95% range) of reported median | 18 (18, 18) | 10 (10, 11) | 18 (18, 18) |
| Median (95% range) of reported lower bound of 95% UR | 9 (9, 10) | 5 (5, 5) | 8 (7, 8) |
| Median (95% range) of reported upper bound of 95% UR | 29 (29, 29) | 18 (18, 19) | 30 (30, 31) |
| Ratio, unnecessary TB treatments per TB death averted |  |  |  |
| Values (median (95% UR)) reported in manuscript | 38 (12, *) | 7.2 (2.3, 43) | 372 (75, *) |
| Median (95% range) of reported median | 38 (38, 39) | 7.3 (7.2, 7.4) | 376 (365, 384) |
| Median (95% range) of reported lower bound of 95% UR | 12 (11, 12) | 2.3 (2.2, 2.3) | 74 (70, 78) |
| Median (95% range) of reported upper bound of 95% UR | Upper bound not defined | 43 (38, 47) | Upper bound not defined |

* Upper bound not defined
