# Supplementary material for: Estimated clinical impact of the Xpert MTB/RIF Ultra cartridge for diagnosis of pulmonary tuberculosis: A modeling study
Source: PLoS Med. 2017 Dec 14;14(12):e1002472. doi: 10.1371/journal.pmed.1002472 (PMC5730108; doi:10.1371/journal.pmed.1002472)
Supplement: S4 Table — The same results are shown graphically in Fig 2. (DOCX) [file pmed.1002472.s010.docx]

**S4 Table: Primary results with different uses of trace call, for all modeled settings (same results are shown graphically in Figure 2)**

|  | Standard Xpert | Ultra without trace | Ultra, conditional trace* | Ultra, positive trace calls repeated* | Ultra with trace |
| --- | --- | --- | --- | --- | --- |
| **TB deaths per 1000 people evaluated** | | | | | |
| India | 10.4 (7.6, 14.3) | 10.2 (7.4, 13.8) | 9.9 (7.3, 13.4) | 10.0 (7.3, 13.6) | 9.9 (7.3, 13.4) |
| South Africa | 15.4 (10.8, 21.2) | 14.5 (10.3, 19.8) | 14.0 (9.9, 18.8) | 14.2 (10.1, 19.2) | 13.9 (9.9, 18.8) |
| China | 2.12 (1.5, 2.9) | 2.10 (1.4, 2.9) | 2.06 (1.4, 2.9) | 2.08 (1.4, 2.9) | 2.06 (1.4, 2.9) |
| **Incremental TB deaths prevented per 1000 people evaluated, compared to standard Xpert** | | | | | |
| India | - | 0.25 (0, 0.9) | 0.46 (0, 1.2) | 0.40 (-0.1, 1.1) | 0.48 (<0,1.3) |
| South Africa | - | 0.83 (0.1, 2.5) | 1.37 (0.3, 3.5) | 1.12 (0.2, 3.1) | 1.42 (0.3, 3.7) |
| China | - | 0.02 (-0.12, 0.16) | 0.05 (-0.08, 0.18) | 0.04 (-0.09, 0.16) | 0.05 (-0.08, 0.19) |
| **Unnecessary TB treatments per 1000 people evaluated** | | | | | |
| India | 56 (38, 80) | 62 (43, 87) | 70 (50, 95) | 67 (48, 92) | 75 (55, 100) |
| South Africa | 363 (227, 497) | 367 (232, 500) | 372 (240, 504) | 369 (234, 502) | 373 (241, 505) |
| China | 17 (10, 25) | 24 (15, 36) | 34 (23, 48) | 27 (17, 40) | 35 (24, 49) |
| **Incremental unnecessary treatments per 1000 people evaluated, compared to standard Xpert** | | | | | |
| India | - | 6 (-1, 14) | 14 (6, 24) | 11 (3, 20) | 18 (10, 29) |
| South Africa | - | 4 (0, 9) | 9 (4, 17) | 6 (1, 12) | 10 (5, 19) |
| China | - | 7 (0, 16) | 17 (8, 29) | 10 (1, 21) | 18 (8, 30) |
| **Unnecessary treatments per TB death prevented** | | | | | |
| India | *-* | 25 (0, **) | 31 (9, 1913) | 27 (6, **) | 38 (12, **) |
| South Africa | *-* | 5 (0, 73) | 7 (2, 38) | 5 (1, 44) | 7 (2, 43) |
| China | *-* | 331 (0, **) | 368 (73, **) | 280 (24, **) | 372 (75, **) |

* In the conditional trace scenario, the trace call is used only for individuals with no previous TB history. In the “positive trace calls repeated” scenario, Ultra is repeated once if the initial result is positive by trace call only; positives (including positives only by trace) on repeat testing are considered positive, while those who are negative on repeat testing are considered negative for TB.

** Upper bound not determined because no deaths were averted in >2.5% of simulations
