# Supplementary material for: Estimated clinical impact of the Xpert MTB/RIF Ultra cartridge for diagnosis of pulmonary tuberculosis: A modeling study
Source: PLoS Med. 2017 Dec 14;14(12):e1002472. doi: 10.1371/journal.pmed.1002472 (PMC5730108; doi:10.1371/journal.pmed.1002472)
Supplement: S5 Table — (DOCX) [file pmed.1002472.s011.docx]

**S5 Table: Re-estimated assay specificities, stratifying study sites by national TB incidence.**

| Lower-TB-incidence settings (<100/100,000 person-years) | No TB history | With TB history |
| --- | --- | --- |
| Specificity of standard Xpert | 99.6% (98.6, 100) | 97.0% (91.6, 99.4) |
| Probability of false positive Ultra (with trace call) if standard Xpert negative | 1.1% (0.7%) ^a^ | 0% ^c^ |
| Probability of false positive Ultra (with trace call) if standard Xpert false-positive | 0% ^d^ | 100% ^c^ |
| Overall specificity of Ultra (with trace call), simulated | 99.1% (97.1, 99.8) ^b^ | 97.0% (91.6, 99.4) ^b^ |
| **Higher-TB-incidence settings (>100/100,000 person-years)** | **No TB history** | **With TB history** |
| Specificity of standard Xpert | 97.6% (95.7, 98.8) | 98.6% (95.2, 99.8) |
| Probability of false positive Ultra (with trace call) if standard Xpert negative | 3.0% (0.8%) ^a^ | 8.2% (2.4%) ^a^ |
| Probability of false positive Ultra (with trace call) if standard Xpert false-positive | 91.0% (10%) ^a^ | 100% ^c^ |
| Overall specificity of Ultra (with trace call), simulated | 94.8% (92.6, 96.6) ^b^ | 90.1% (84.5, 94.1) ^b^ |

^a^ Mean (standard deviation) of beta distribution

^b^ Median (95% uncertainty range) of simulated parameter values

^c^ Assumed result same as standard Xpert due to full concordance of the limited data available.

^d^ No range modeled, because only one (Ultra-negative) subject contributed to this estimate.
