# Supplementary material for: Estimated clinical impact of the Xpert MTB/RIF Ultra cartridge for diagnosis of pulmonary tuberculosis: A modeling study
Source: PLoS Med. 2017 Dec 14;14(12):e1002472. doi: 10.1371/journal.pmed.1002472 (PMC5730108; doi:10.1371/journal.pmed.1002472)
Supplement: S6 Table — (DOCX) [file pmed.1002472.s012.docx]

**S6 Table: Reduction in untreated rifampin-susceptible and rifampin-resistant TB cases when using Ultra**

|  |  | # of cases per 1000-person cohort | % of cases not properly treated, standard Xpert* | % of cases not properly treated, Ultra* | Difference in cases not properly treated, per 1000-person cohort* |
| --- | --- | --- | --- | --- | --- |
| **India** | **DS-TB** | 111 | 10% (7, 14) | 7% (4, 11) | 3.4 (0.7, 6.2) |
|  | **RR-TB** | 7 | 16% (11, 21) | 15% (10, 21) | 0.04 (-0.1, 0.2) |
| **South Africa** | **DS-TB** | 113 | 14% (9, 20) | 6% (2, 10) | 8.9 (4.3, 14.7) |
|  | **RR-TB** | 5 | 28% (20, 35) | 21% (14, 29) | 0.3 (0, 0.6) |
| **China** | **DS-TB** | 58 | 11% (7, 15) | 8% (4, 12) | 1.6 (0.1, 3.1) |
|  | **RR-TB** | 5 | 15% (10, 21) | 15% (10, 21) | 0.02 (-0.10, 0.14) |

*median (95% uncertainty range). Cases not properly treated include rifampin-susceptible TB cases who were not treated for TB, and rifampin-resistant cases who were not treated or were treated with first-line therapy. DS=drug susceptible; RR=rifampin-resistant.
