# Supplementary material for: Estimated clinical impact of the Xpert MTB/RIF Ultra cartridge for diagnosis of pulmonary tuberculosis: A modeling study
Source: PLoS Med. 2017 Dec 14;14(12):e1002472. doi: 10.1371/journal.pmed.1002472 (PMC5730108; doi:10.1371/journal.pmed.1002472)
Supplement: S7 Table — (DOCX) [file pmed.1002472.s013.docx]

**S7 Table: Results with more pessimistic estimates of future rifampin-resistant TB treatment outcomes**

|  | Standard Xpert,  median (95% UR) | Ultra,  median (95% UR) | | Difference (or ratio of differences), Ultra vs standard Xpert,  median (95% UR) [80% UR] |
| --- | --- | --- | --- | --- |
| **TB deaths** |  |  | |  |
| Indian TB clinic | 11.5 (8.6, 15.4) | 11.0 (8.3, 14.7) | | -0.46 (-1.2, 0.0) [-0.9, -0.2] |
| South African HIV clinic | 15.8 (11.0, 21.8) | 14.4 (10.1, 19.2) | | -1.36 (-3.7, -0.2) [-2.8, -0.5] |
| Chinese primary care clinic | 2.84 (2.1, 3.7) | 2.79 (2.1, 3.6) | | -0.04 (-0.2, 0.1) [-0.1, 0] |
| **Unnecessary TB treatments** | | | |  |
| Indian TB clinic | 56 (38, 80) | 75 (55, 100) | | 18 (10, 29) [13, 25] |
| South African HIV clinic | 363 (229, 497) | 374 (242, 505) | | 10 (5, 19) [7, 15] |
| Chinese primary care clinic | 17 (10, 25) | 35 (24, 48) | | 18 (8, 30) [11, 26] |
| **Unnecessary treatments per TB death averted** | | | |  |
| Indian TB clinic | *-* | | - | *40 (13, *) [18, 125]* |
| South African HIV clinic | *-* | | - | *7.6 (2.3, 49) [3.4, 21]* |
| Chinese primary care clinic | *-* | | - | *372 (75, *) [130, *]* |

* Upper bound not determined because more deaths occurred with Ultra than with standard Xpert in >2.5% (or for 80% UR, >10%) of simulations
