# Supplementary material for: Estimated clinical impact of the Xpert MTB/RIF Ultra cartridge for diagnosis of pulmonary tuberculosis: A modeling study
Source: PLoS Med. 2017 Dec 14;14(12):e1002472. doi: 10.1371/journal.pmed.1002472 (PMC5730108; doi:10.1371/journal.pmed.1002472)
Supplement: S8 Table — (DOCX) [file pmed.1002472.s014.docx]

**S8 Table: Results for Chinese primary care setting, with and without empiric treatment***

|  | Standard Xpert | Ultra | Difference (or ratio of differences), Ultra vs standard Xpert |
| --- | --- | --- | --- |
| **TB deaths, China** |  |  |  |
| Original parameter estimates | 2.12 (1.5, 2.9) | 2.06 (1.4, 2.9) | -0.05 (-0.2, 0.1) |
| Revised parameter estimates* | 2.12 (1.5, 2.9) | 2.06 (1.4, 2.9) | -0.05 (-0.2, 0.1) |
| **Unnecessary TB treatments** | | |  |
| Original parameter estimates | 17 (10, 25) | 35 (24, 49) | 18 (8, 30) |
| Revised parameter estimates* | 59 (39, 84) | 76 (55, 103) | 17 (7, 29) |
| **Unnecessary treatments per TB death averted** | | |  |
| Original parameter estimates | *-* | - | *372 (75, **)* |
| Revised parameter estimates* | *-* | - | *367 (72, **)* |

* Revised parameter estimates include 4% [range 2-8%] empiric treatment in Chinese primary care setting, as estimated for Indian TB center setting in primary analysis

** Upper bound not determined because more deaths and/or fewer unnecessary treatments occurred with Ultra than with standard Xpert in >2.5% of simulations
