# Supplementary material for: Estimated clinical impact of the Xpert MTB/RIF Ultra cartridge for diagnosis of pulmonary tuberculosis: A modeling study
Source: PLoS Med. 2017 Dec 14;14(12):e1002472. doi: 10.1371/journal.pmed.1002472 (PMC5730108; doi:10.1371/journal.pmed.1002472)
Supplement: S9 Table — (DOCX) [file pmed.1002472.s015.docx]

**S9 Table: Results with altered clinical decision-making based on knowledge of Ultra’s different test characteristics**

|  | Original model | Chest x-ray as confirmatory test when using Ultra^§^ | | 20% reduction in empiric treatment after negative Ultra in HIV clinic cohort |
| --- | --- | --- | --- | --- |
| Indian TB clinic | -0.48 (-1.3, 0.0) | +0.37 (-1.1, +0.2) | |  |
| South African HIV clinic | -1.42 (-3.7, -0.3) | -0.39 (-1.8, +0.4) | | -1.27 (-3.5, -0.1) |
| Chinese primary care clinic | -0.05 (-0.2, 0.1) | +0.10 (-0.06, +0.27) | |  |
| **Difference in unnecessary TB treatments, Ultra versus Xpert** | | | |  |
| Indian TB clinic | 18 (10, 29) | 1 (-5, +8) | |  |
| South African HIV clinic | 10 (5, 19) | 0 (-4, +5) | | -57 (-87, -27) |
| Chinese primary care clinic | 18 (8, 30) | 0 (-6, +7) | |  |
| **Ratio, Unnecessary treatments per TB death averted** | | | |  |
| Indian TB clinic | 38 (12, *) | | † |  |
| South African HIV clinic | 7.2 (2.3, 43) | | ‡ | # |
| Chinese primary care clinic | 372 (75, *) | | † |  |

§ CXR modeled as having 90% sensitivity for pulmonary TB in individuals with HIV [9,10], 95% sensitivity in HIV-uninfected individuals [7,8], and 50% specificity [11,12].

* Upper bound not determined because more deaths occurred with Ultra than with standard Xpert in >2.5 of simulations

† Ratio not calculated because combined (Ultra + confirmatory test) algorithm is not expected to avert TB deaths.

‡ Ratio not calculated because combined algorithm results in small but inconsistent mortality benefit and minimal expected difference in unnecessary treatments.

# Ratio not calculated because this use of Ultra resulted in both fewer deaths and fewer unnecessary treatments.
