# Supplementary material for: Estimated clinical impact of the Xpert MTB/RIF Ultra cartridge for diagnosis of pulmonary tuberculosis: A modeling study
Source: PLoS Med. 2017 Dec 14;14(12):e1002472. doi: 10.1371/journal.pmed.1002472 (PMC5730108; doi:10.1371/journal.pmed.1002472)
Supplement: S10 Table — (DOCX) [file pmed.1002472.s016.docx]

**S10 Table: Results in hypothetical scenario of an imperfect reference standard as the cause of some positive Ultra results**§

|  | Indian TB center | South African HIV clinic | Chinese primary care |
| --- | --- | --- | --- |
| **TB prevalence in cohort in primary analysis, per 1000** | 117.6 | 117.6 | 62.5 |
| **True TB prevalence within cohort under 95% culture sensitivity assumption, per 1000** | 123.8 | 123.8 | 65.8 |
| **Cases detected by culture and Ultra, per 1000** | 108.2 | 105.5 | 57.3 |
| **Cases detected by culture and not Ultra, per 1000** | 9.4 | 12.1 | 5.2 |
| **Cases detected by Ultra but not culture, per 1000** | 3.1 | 3.1 | 1.6 |
| **Cases missed by both culture and Ultra, per 1000** | 3.1 | 3.1 | 1.6 |
| **Estimated deaths prevented among additional cases detected by Ultra and not culture, per 1000*** | 0.42 | 0.29 | 0.05 |
| **Revised deaths averted per 1000** | 0.91 | 1.72 | 0.11 |
| **Revised unnecessary treatments per 1000** | 15 | 7 | 16 |
| **Revised ratio, unnecessary treatments per death averted** | 16 | 4 | 103 |
| **Original (median) ratio, unnecessary treatments per death averted** | 38 | 7 | 372 |

§ Assumes 5% of cases were missed by culture, and half of those culture-negative cases were detected by Ultra

* Uses median simulated value from primary analysis, for death averted per additional case detected by Ultra relative to standard Xpert: 0.14 in India, 0.09 in South Africa, and 0.03 in China.
